# Bursting noise in gene expression dynamics: linking microscopic and mesoscopic models

**DOI:** 10.1098/rsif.2015.0772

**Published:** 2016-01

**Authors:** Yen Ting Lin, Tobias Galla

**Affiliations:** Theoretical Physics, School of Physics and Astronomy, The University of Manchester, Manchester M13 9PL, UK

**Keywords:** bursting noise, piecewise deterministic Markov process, stochastic processes, first passage times, weak noise limit, coarse-grained models

## Abstract

The dynamics of short-lived mRNA results in bursts of protein production in gene regulatory networks. We investigate the propagation of bursting noise between different levels of mathematical modelling and demonstrate that conventional approaches based on diffusion approximations can fail to capture bursting noise. An alternative coarse-grained model, the so-called piecewise deterministic Markov process (PDMP), is seen to outperform the diffusion approximation in biologically relevant parameter regimes. We provide a systematic embedding of the PDMP model into the landscape of existing approaches, and we present analytical methods to calculate its stationary distribution and switching frequencies.

## 1. Introduction

Transcription and translation in the process of gene expression occur at the molecular level and in environments of relatively small copy numbers. The discreteness of the molecular dynamics and the inherent randomness with which reactions occur are known as ‘intrinsic noise’. It is now widely accepted that intrinsic noise plays an important role in gene regulatory networks [1–3]. It promotes epigenetic diversity and enhances the adaptability of a single phenotype in changing environments [4,5]. To investigate the effects of intrinsic noise, mathematical models at different levels have been constructed, ranging from microscopic models [3,6–10] describing the finer origins of intrinsic noise to mesoscopic models [11–15]. While the former capture the biological processes in more detail, the latter are computationally scalable and constructed to model more complex networks. These models all capture some signatures of intrinsic noise, but the detailed implementation of stochasticity varies from model to model. It is then important to consider how noise propagates between different levels of mathematical modelling. At present, coarse-grained models are often proposed *ad hoc* and not derived from the more detailed lower scale models. Is this always mathematically appropriate? What statistics of noise should modellers use at different levels of coarse graining? What are the consequences of the choice of noise statistics, and what are the pitfalls in deriving models on the meso-level from finer models on smaller scales? These are some of the questions we aim to address in this work.

The above difficulties in transitioning between different levels of modelling can nicely be illustrated in the context of biological switches. These are systems with different metastable states and the possibility to ‘switch’ between those states. Biological organisms with such behaviour include the *Lac* switch [6] in *Escherichia coli* and the *Enterobacteria phage λ* switch [7]. Computational and mathematical models of these range from very detailed descriptions [6,7] over individual-molecule approaches [8–10,16] to mesoscopic models [11,12,14,15].

The difficulties in connecting these different levels of modelling biological switches are amplified by the recent recognition that the mRNA populations are essential to describing the statistics of regulatory processes [16,17]. Biologically, mRNA molecules are a relatively short-lived source compared to the proteins into which they ultimately translate. Protein production from a given mRNA molecule proceeds while it exists, but ceases after the mRNA decays. This leads to a production of protein in bursts—that is, the production is active for a relatively short and random period of mRNA lifetime, and during that time a *random number* of proteins is generated. This phenomenon is termed *translational bursting* [1] and it can be observed in single-molecule experiments [18]. While some mesoscopic models account for such bursting [14,15], the theoretical investigation of these processes is often limited to their stationary distribution and frequently does not include dynamic features such as switching times.

The aim of our work is to investigate the effects of bursting noise in gene regulatory networks [9,10,11,13,16,19–21], and to construct connections between individual-based models and mesoscopic approaches. Specifically, we start from microscopic and individual-molecule-based models of a toggle switch and set out to construct coarse grained, mathematically tractable models without systematically biasing the outcomes.

## 2. Models of a toggle switch

### 2.1. Different scales of individual-based models

**Figure 1.**
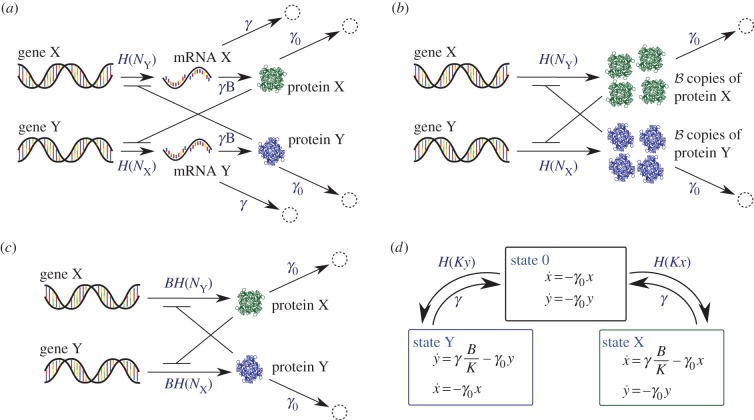
Schematic diagrams illustrating the model dynamics. (*a*) Full model (FM) describing both the mRNA and the protein populations. (*b*) Protein-only model with geometrically distributed (GB) or constant (CB) bursts. The quantity 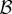 is a geometrically distributed random number with mean *B* in the GB model, and 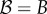 is a constant in the CB model. (*c*) Protein-only model without bursts (NB). (*d*) The piecewise deterministic Markov process (PDMP). (Online version in colour.)

We compare four individual-based models and investigate the effect of bursting noise in a toggle switch network. The first model we consider describes both the mRNA and the protein population dynamics [16]. Figure 1*a* illustrates the Markovian model of the regulatory network. Genes X and Y are transcribed into mRNA X and mRNA Y, respectively, which in turn are translated to produce proteins X and Y. The transcription of each of the two genes is suppressed by proteins of the respective other type via a Hill function [2,3] 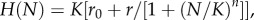 where *N* stands for the number of suppressing proteins. The model parameter *K* represents a typical population scale of the proteins, and the parameters *r* and *r*_0_ set the minimal (*r*_0_*K*) and maximal transcription rates (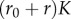). The parameter *n* > 0 is the so-called Hill coefficient which models the cooperative binding of the repressors [3]. More details of the reaction scheme can be found in the electronic supplementary material. Proteins of either type, and the mRNA molecules degrade with constant rates *γ*_0_ and *γ*, respectively. Biologically, mRNA molecules degrade much faster than the proteins do (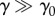) [2,14,18]. The translation rate of the mRNA is parametrized by *γB*, where the parameter *B* is the relative frequency of protein production to mRNA degradation. In this parametrization, the number of proteins one single mRNA molecule produces during its lifetime is a geometrically distributed random variable with mean *B* (see the electronic supplementary material). Biologically, the parameter *B* varies depending on the type of product protein [22]. We assume 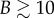 in this work [2,8] to investigate the effect of translational bursting. Together with the relatively short lifetime of mRNA molecules, this constitutes the origin of ‘translational bursting’ in the model [14,23]: a relatively large number of protein molecules is synthesized in a relatively short period of time.

For simplicity, the process in figure 1*a* is assumed to be symmetric with respect to X and Y, but the analysis is easily generalized to asymmetric circuits. In table 1, we list a set of estimated values of the parameters for the model organism *E. coli*, along with relevant references. While the parameters of our model are expressed in time units of cell cycles, it is important to keep in mind that the model does not explicitly capture cell reproduction and cell division.

**Table 1.** Parameter set.

parameter	description	value	unit	references
*B*	average number of proteins each mRNA produces	30	molecule	[2]
*γ*	mRNA degradation rate	30	1/(cell cycle)	[2]
*γ*_0_	protein degradation rate	1.0	1/(cell cycle)	[2,9,24]
*r*	maximum suppressed transcription rate	6/100	1/(cell cycle)	[13,25]^a^
*r*_0_	basal transcription rate	1/150	1/(cell cycle)	[13,25]^b^
*K*	a typical population scale of the proteins	200	molecule	[13,25]^c^
*n*	Hill coefficient	3.0	dimensionless	[9,13,19,25]

^a^In [13], *r* = 1.8 and the time unit is defined as the inverse of the protein degradation rate. In the FM, we use this value, normalized by the mean burst size *B* = 39 molecules (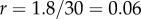).

^b^In [13], *r*_0_ = 0.2. After normalizing with respect to the burst size 30, we obtain 1/150. In [25], *r*_0_ = 0.05*r*, which is of the same order as [13].

^c^In [13], *K* is set to be 200 molecules. In [25], only the deterministic dynamics are provided and 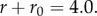 To match the protein population scale ≈400 in [13,24], we impose *rK* = 400, resulting in a typical population scale of the proteins *K* ∼ 100 molecules, which is of the same order as that of Lu *et al*. [13].

In the context of this work, the model just described constitutes the most detailed model we will investigate and compare against. It serves as a starting point for the derivation of more coarse-grained models, and for these purposes we will refer to it as the ‘full model’ (*FM*) in the following. The FM is of course a simplified model itself, which does not describe the full complexity of the underlying biology. For example, it does not capture further genetic states accessed at a faster time scale, or dimerization processes which happen further downstream in the regulatory circuit [3,26]. We refer to it as the ‘FM’ solely to indicate that it is the most detailed model within the remit of this work.

The FM describes both the mRNA and the protein populations, hence it constitutes a relatively high-dimensional system which complicates the mathematical analysis. Notably, the only role of mRNA in the FM is to generate proteins, and so mRNA can be left out, so long as the correct statistics of protein production is retained. The timescale separation between the mRNA and protein lifetimes leads to the following reduced model describing only the protein dynamics. In the limit of infinitely fast mRNA degradation (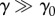), proteins are generated instantaneously in bursts of geometrically distributed sizes with a mean *B*, and in between bursting events protein populations decay with rate *γ*_0_. We will refer to the reduced model as the *GB* model (geometrically distributed bursts) (figure 1*b*; [17,22]). In the GB model, the transcription rates are regulated via the Hill function exactly as before in the FM.

A further reduction of the GB model involves replacing the GB sizes by a constant size *B*. We will call this the *CB* model (constant bursts) [8]. While the reduction of the FM to the model with geometrically distributed bursts is well controlled and exact in the limit 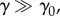 the effects of introducing CB sizes are unclear at this stage and require a detailed analysis (see below).

An even more reduced model is a model with no bursts [8–10], we will refer to this as the *NB* model. The reaction scheme is illustrated in figure 1*c*. In this model, only one single protein is synthesized when a transcription event occurs. We assume a *B*-fold increased transcription rate so that the average number of proteins synthesized per unit time is consistent with the FM, GB and CB models.

### 2.2. Stationary distributions

**Figure 2.**
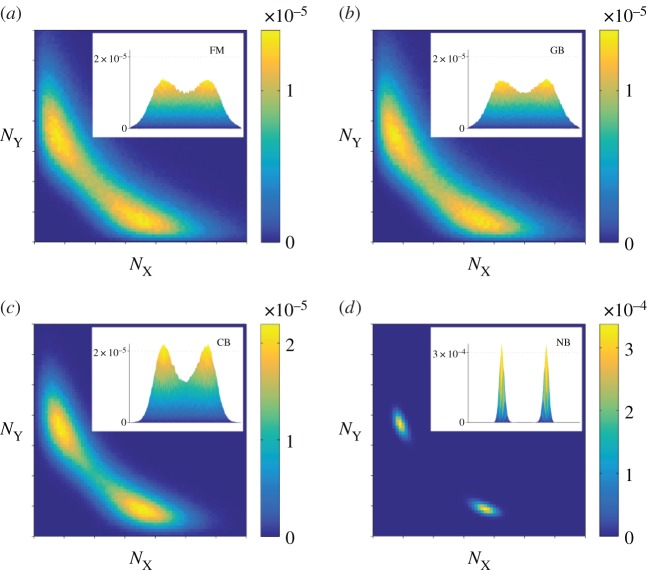
Stationary distribution of protein numbers, shown in the range 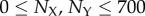 on a linear scale on both axes. (*a*) FM: full model describing the mRNA and protein populations; (*b*) GB: protein-only model with geometrically distributed bursts; (*c*) CB: protein-only model with constant bursts; and (*d*) NB: protein-only model without bursts. (Online version in colour.)

Numerical simulations of each of the models are carried out using standard methods [27,28]. In the following, we present statistical properties of the models, leaving typical sample paths to the electronic supplementary material. Figure 2 displays the numerically computed stationary distributions for the FM, GB, CB and NB models. In this section, we discuss the outcomes of the different models qualitatively. A more quantitative comparison of the stationary distributions can be found in the electronic supplementary material.

The data in the figure illustrate that the profiles of protein expressions in different model settings are quite distinct. This is due to the different representations of the underlying intrinsic noise. While the stationary distributions of the FM and the GB model are in good agreement with each other, substantial discrepancies from the FM are found in the CB and NB models. In the CB model, the stationary distribution of protein numbers is very localized compared with the FM and the GB model. In the NB model, the probability distribution is even more sharply concentrated. This is because the NB model misses out two pertinent sources of noise. Bursting production in the CB model amplifies the stochasticity of transcription events and leads to a broadening of the protein distribution. Adding randomly distributed burst sizes (GB model) introduces further stochasticity and diversifies protein numbers even further. Based on these results, we conclude that the bursting noise introduced by the mRNA populations significantly broadens the stationary distribution. In addition, the GB model approximates the FM model significantly better than the CB and NB models do. We can effectively discard the CB and NB models as faithful representations of the FM, and our subsequent discussion hence focuses mostly on the GB model.

### 2.3. Mean first switching time

The toggle switch has two dynamic attractors, one in which protein X is highly expressed and where protein Y has a low concentration, and the other with inverted roles by symmetry. Starting from one attractor, the switch can be driven to the other attractor by fluctuations. The timescale of such a transition quantifies the dynamical stability: the longer the timescale, the more stable the system is at the initial position. As we will study next, the way in which the bursting production of protein is implemented significantly affects the timescale of these switching processes.

Starting from initial condition 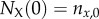 and 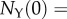 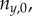 we define the first switching time as the time it takes for a sample path to reach the symmetric boundary 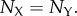 Mathematically, the first switching time is a random variable. The mean first switching time (MFST) is then the average value of the random first switching time. The MFST depends on the initial condition 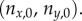


**Figure 3.**
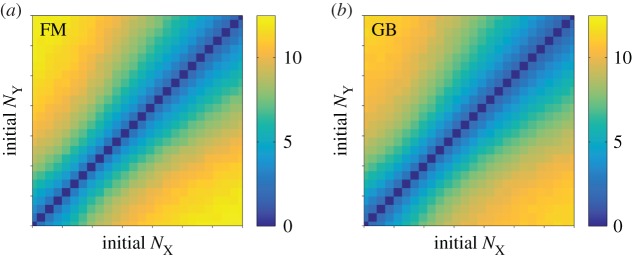
MFST as a function of the initial protein numbers (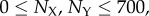 shown on a linear scale). (*a*) FM: full model; (*b*) GB model. (Online version in colour.)

Sweeping across the space of possible initial configuration, the MFST of the FM and of the GB model are measured in simulations and presented in figure 3. We show the MFST of the CB in the electronic supplementary material. As with the stationary distributions, the data in figure 3 indicate that the GB model approximates the switching times of the FM to a good accuracy. A more detailed quantitative comparison is again provided in the electronic supplementary material. We remark that the MFST of the CB model is almost twice as long as that of the GB and FM models, and the switching time in the NB model is longer than 1000 cell cycles (see the electronic supplementary material).

### 2.4. Diffusion approximation

The evolution of the protein population in the GB model is described by a master equation (see the electronic supplementary material). Solving master equations mathematically is however difficult and mostly limited to linear dynamics [22,29]. The only realistic way forward for a theoretical analysis is often the so-called diffusion approximation.

In the diffusion approximation, the discrete-molecule process is approximated by a Gaussian process for continuous concentrations—numbers of the different types of molecules normalized by a typical population scale. The Gaussian process satisfies a diffusion equation (the Fokker–Planck equation) [30,31]. Based on these methods, it is often possible to calculate or approximate the stationary behaviour and switching times of model gene networks. For existing studies in the context of toggle switches, see [11–13].

Deriving the diffusion approximation of the GB model requires modest modifications to the standard Kramers–Moyal expansion [30,31]. These modifications are necessary to account for the randomness induced by the GB size. Details of the derivation can be found in the electronic supplementary material, we here only report the final outcome. The expansion results in two coupled Itō stochastic differential equations for the concentrations 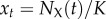 and 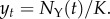 These are valid in the limit of large but finite populations [32] and they are of the form
2.1*a*
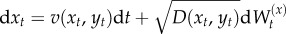
and
2.1*b*
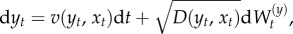
with drift *v* and diffusion *D* given by
2.2*a*
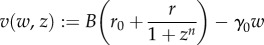
and
2.2*b*
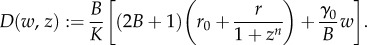
The quantities 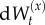 and 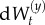 represent independent Wiener processes.

The diffusion approximation can only be expected to be accurate when molecule numbers are large so that the concentations *x_t_* and *y_t_* are effectively continuous. In principle, a similar analysis can also be applied to the master equations of the FM. In the FM, mRNA numbers are rather small though (typically less than 5, see the electronic supplementary material), so the Gaussian approximation does not capture the statistics of the intrinsic noise faithfully. Similarly, a further analysis of the CB and NB models can be carried out based on the diffusion approximation. Given that CB and NB models fail to reproduce the behaviour of the FM, these results are relegated to the electronic supplementary material.

**Figure 4.**
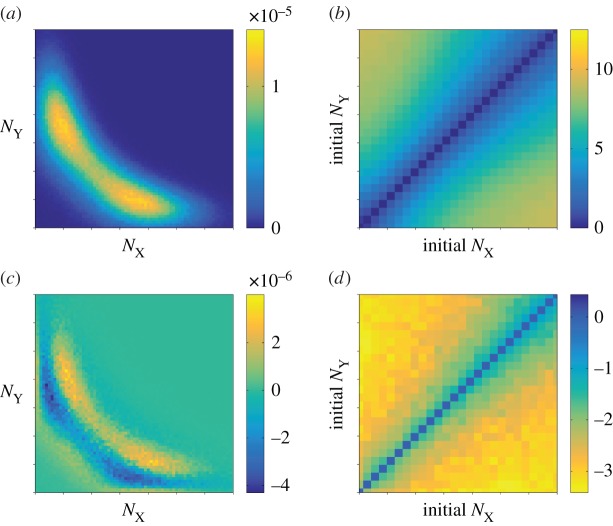
Diffusion approximation of the protein-only model with geometrically distributed random bursts (GB). (*a*) Stationary distribution as a function of the protein numbers. (*b*) Mean first switching time (MFST) as a function of the initial protein numbers, in the unit of cell cycles. (*c*) Net deviation of the stationary distribution from the full model. (*d*) Net deviation of the MFST from the FM. All axes show the range 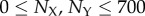 on linear scales. (Online version in colour.)

Results from simulating the Gaussian process of equations (2.1*a*,*b*) are shown in figure 4. While the data for the stationary distribution (figure 4*a*) looks similar to that of the FM (figure 2*a*), notable discrepancies are manifest in the MFSTs (cf. figure 4*b* and figure 3*a*). In figure 4*c*,*d*, we show the differences between simulation outcomes of the FM and those of the diffusion approximation of the GB model. Although the GB model itself approximates the FM well (figures 2 and 3), we conclude that the diffusion approximation fails to capture the relevant model outcomes.

## 3. Construction of a new mesoscopic model

### 3.1. Piecewise deterministic Markov process

We have seen that the diffusion approximation of the GB model fails to reproduce the statistics of the FM. This underlines the need to construct coarse-grained models *directly from the FM* and without the intermediate step of a protein-only dynamics. We now proceed to introduce such a model. As before, we describe protein concentrations by continuous variables, *x* and *y*. The mRNA dynamics are captured by introducing three states: the 0-state describes phases in which no mRNA is present. In the X-state, there is one mRNA of type X and protein X is generated with rate *γb*. The quantity *b* = *B*/*K* is the mean burst size in the unit of protein concentration. No proteins of type Y are produced in the X-state. Similarly, in the Y-state, protein Y is generated with rate *γb*. Both types of protein are subject to natural degradation with rate *γ*_0_ in any of the three states.

This is described by the following *deterministic* differential equations:
3.1*a*
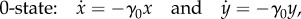

3.1*b*
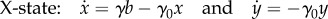

3.1*c*
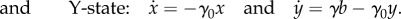
The rates with which the system transits between the states are based on the dynamics of the FM:
3.2
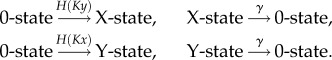
No transitions occur directly between the X- and Y-states. The kinetic scheme is illustrated in figure 1*d*.

The stochasticity and discreteness of the mRNA populations is reflected in the random transitioning between the 0-, X- and Y-states. Between these Markovian events, the protein concentrations evolve deterministically. We will refer to this model as the piecewise deterministic Markov process (PDMP).

Notably, at most one mRNA molecule of either type can be present in the PDMP at any time. Although the model can be generalized to allow more than one mRNA molecule, the analysis below shows that the lowest order approximation is sufficient to capture the relevant fluctuations of the mRNA dynamics.

### 3.2. Performance of mesoscopic models

**Figure 5.**
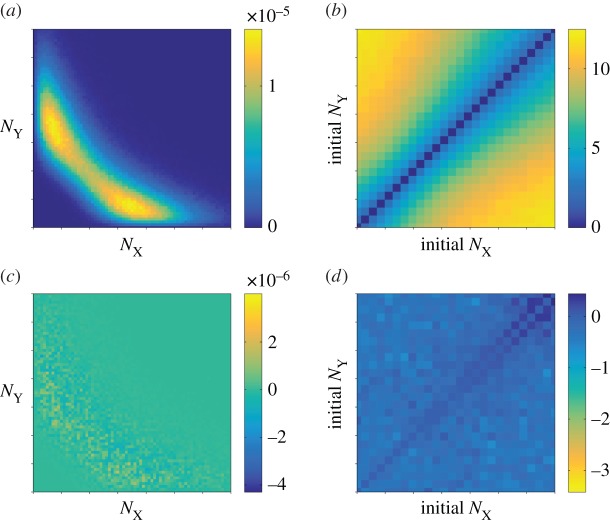
PDMP approximation. (*a*) Stationary distribution. (*b*) MFST in the unit of cell cycles as a function of initial protein numbers. (*c*) Net deviation of the stationary distribution from the full model. (*d*) Net deviation of the MFST of the PDMP model from the FM. All axes are on linear scales and show the range 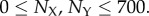 (Online version in colour.)

As in the GB model, we work in the limit of infinitely fast-degrading mRNA (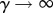). Simulations of the PDMP model in this limit can be carried out using a minor modification of a previously proposed algorithm [15]. We measure the stationary distribution of the PDMP model and the MFSTs for different initial protein numbers. Results are shown in figure 5*a*,*b*, and we compare the outcome against that of the FM in figure 5*c*,*d*.

The simulation data indicate that the PDMP approximation outperforms the diffusion approximation of the GB model, and it provides a more faithful approximation to the FM. This is because the diffusion approximation introduces Gaussian noise. It retains some information about the variance of protein production and degradation, but it does not capture the geometrically distributed burst sizes in the GB model well enough. The PDMP approximation, on the other hand, models exponentially distributed bursts in protein concentration. The exponential distribution in the PDMP model is the analogue of the geometric distribution in the discrete-molecule GB model. While the PDMP model is an approximation as well, it retains the typical characteristics of the stationary distribution and switching times of the original model. At the same time, the PDMP model is suitable for further mathematical analysis (see below).

**Figure 6.**
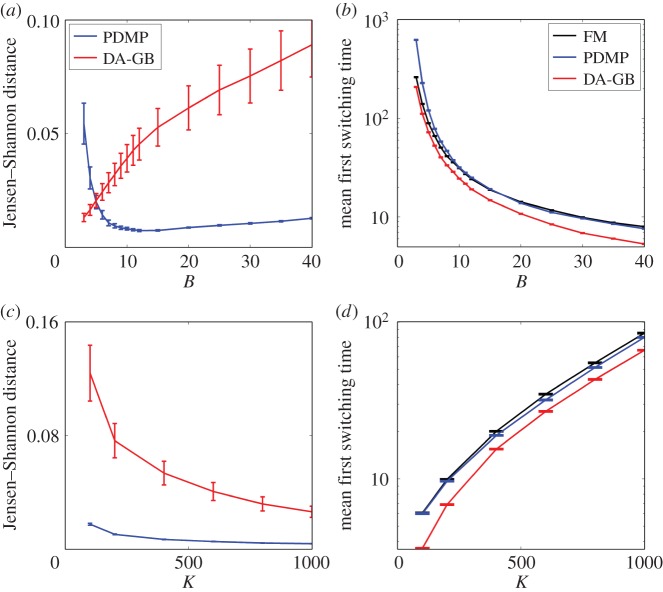
Performance of the PDMP model and the diffusion approximation of the GB model (DA-GB). (*a*) Jensen–Shanon distance between the stationary distribution PDMP (and DA-GB) and the stationary distribution of the FM. (*b*) MFST for varying value of *B* at fixed *K* = 200. (*c*,*d*) Similar to (*a*,*b*) but now varying *K* at fixed *B* = 30. (Online version in colour.)

We now investigate the robustness of these findings. In figure 6, we vary two essential parameters, the mean burst size *B* and the population scale *K*, while keeping the other parameters fixed. We measure the Jensen–Shannon distance [33,34] between the resulting stationary distributions of the PDMP and that of the FM. Data are shown in figure 6*a*,*c*. We also compare the MFSTs starting from one of the stable modes (figure 6*b*,*d*). The figures also show results from the diffusion approximation of the GB model.

Results indicate that the PDMP model outperforms the diffusion approximation of the GB model for mean burst sizes of 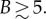 We conclude that the bursting noise has to be considered in this biologically relevant regime [22]. The PDMP model incorporates only the bursting noise and neglects the demographic noise from random degradation of the proteins. The strength of this demographic noise is proportional to 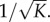 The results in figure 6*c*,*d* indicate that the difference in describing intrinsic noise propagates to physical observables even when the noise is weak (*K* ≈ 1000 for fixed *B* = 30).

## 4. Analytical investigations of the piecewise deterministic Markov process model

### 4.1. Forward equation

The simplicity of the PDMP approach allows us to proceed with a mathematical analysis. We here only outline the main steps, further details are reported in the electronic supplementary material. We denote the probability density that the system is in the 0-state and with protein densities *x*, *y* at time *t* by 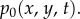 Similarly, we write 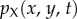 and 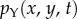 when the system is in the X- or Y-states. The evolution of these distributions then follows the following forward equation
4.1
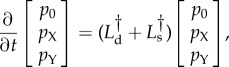
where 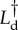 and 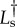 drive the deterministic flow and the random switching between states, respectively. These operators are of the form
4.2*a*
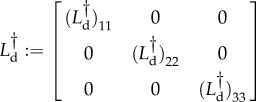
and
4.2*b*
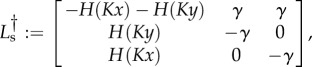
with
4.3*a*
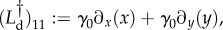

4.3*b*
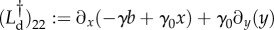

4.3*c*
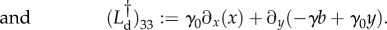
The differential operators 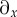 and 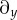 act on all that follows to their right, including the probability densities 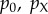 and *p*_Y_ outside the matrix notation in equations (4.1) and (4.2).

The PDMP approximation applies in the limit 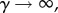 i.e. for fast return into the 0-state. The resident time in the X- and Y-states is exponentially distributed and scales as *γ*^−1^. It formally tends to zero as 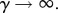 On the other hand, the translation rate *γB* tends to infinity in this limit. Combining the limiting behaviours of resident time and translation rate results in an *exponentially distributed* increment of protein concentration in each cycle of switching from the 0-state to the X- or Y-state, and then returning to the 0-state. As a consequence, the PDMP converges to previously proposed continuous-state bursting models [14,15,18] in the limit 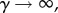 and 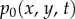 satisfies
4.4
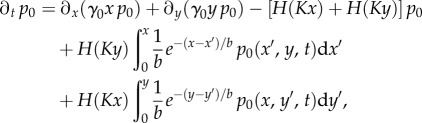
as detailed in the electronic supplementary material.

### 4.2. Mean first switching time

One of the strengths of the PDMP formulation (equations (4.1) and (4.2)) is the relative ease with which MFSTs can be obtained. We first proceed by computing mean escape time from an arbitrary open domain *Ω*. The MFST can be calculated by setting 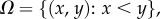 recognizing that the process can only exit this domain by crossing the boundary *x* = *y*.

Suppose, the system is initially at 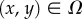 and in state 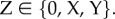 We write 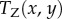 for the mean first time at which the process exits the domain *Ω*. The quantities *T*_z_ then satisfy the following backward equation [30,31]
4.5
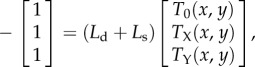
where *L*_d_ and *L*_s_ are adjoint to the operators in equations (4.2). They are given by
4.6*a*
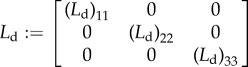
and
4.6*b*
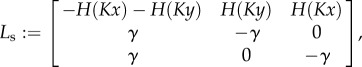
with
4.7*a*
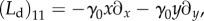

4.7*b*
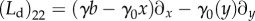

4.7*c*
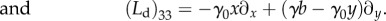
The backward operator (4.6) can be derived either by one-step conditioning [30] or formally using Dynkin's formula [35]. In the infinitely fast-degrading mRNA limit (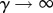), and using appropriate boundary conditions (see the electronic supplementary material), we arrive at
4.8
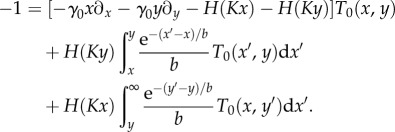


**Figure 7.**
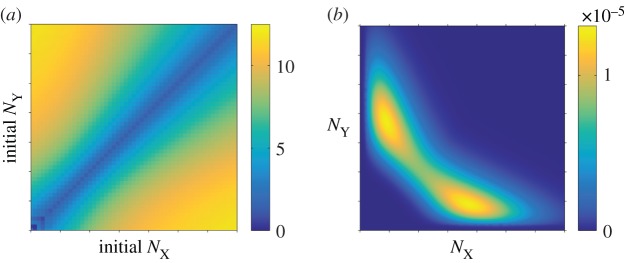
Theoretical prediction of the PDMP model. (*a*) Mean first passage time as a function of initial protein numbers, calculated from the backward equation 4.8). (*b*) Stationary distribution of protein numbers calculated from the WKB method. Axes of both panels show the range 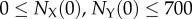 on linear scales. (Online version in colour.)

This is the adjoint equation [30] of the expression in equation (4.4) on the open domain *Ω*. Equation (4.8) is solved by a finite-difference method (see the electronic supplementary material), noting that it is self-consistent and no boundary condition needs to be specified. The solution is shown in figure 7 and reproduces the simulation outcome of the FM well.

We remark that equation (4.8) is only valid for the half-plane *Ω*. A detailed discussion can be found in the electronic supplementary material.

### 4.3. Stationary distribution in the weak-noise limit

**Figure 8.**
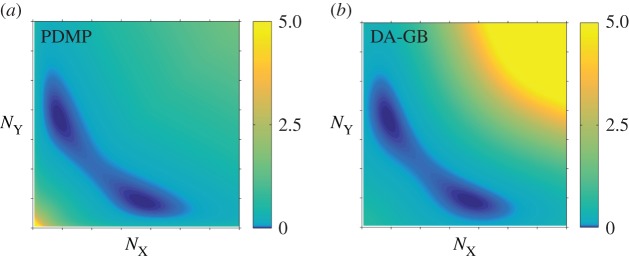
Quasi-potential *S*_0_ as function of the protein numbers 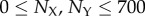 on a linear scale. (*a*) PDMP model. (*b*) Diffusion approximation of the GB model. (Online version in colour.)

The analytical calculation of the stationary distributions of the PDMP model can be pursued further using the so-called Wentzel–Kramers–Brillouin (WKB) method. This technique is based on the ansatz
4.9
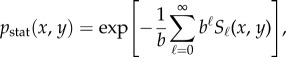
where 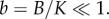 One proceeds by considering 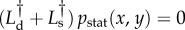 order-by-order in *b*. To leading order, we find the Hamilton–Jacobi equation
4.10
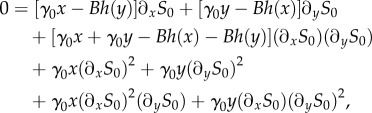
where 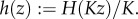 This equation is then numerically solved using the algorithm of Heymann and Vanden–Eijnden [36]. Results are shown in figure 8. Even though this only provides a first-order approximation and despite the fact that we have used *b* = 0.15 (which is not very small), we obtain a reasonable agreement with the stationary distribution in figure 5*a*.

For completeness, we have also carried out a WKB analysis of the diffusion approximation of the GB, CB and NB models. These are presented in the electronic supplementary material.

The leading-order function *S*_0_(*x*, *y*) is the quasi-potential with respect to the stable fixed point, and it quantifies the rare-event statistics of the process in the weak-noise limit 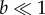 [37,38]. Several studies have suggested that *S*_0_(*x*, *y*) is a suitable candidate for a ‘landscape’ of the non-equilibrium random processes in models of gene regulatory networks [9–11,16,17]. The Hamilton–Jacobi equation (4.10) contains cubic terms such as 
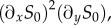
 while diffusion equations are quadratic in the derivatives of *S*_0_. This illustrates the fundamental difference between the statistics of intrinsic noise in the diffusion approximation and the bursting noise in the PDMP. Further more rigorous mathematical investigations into these differences would be very welcome in our view.

We compare the functions *S*_0_ of the PDMP and the diffusion approximation of the GB model in figure 8. One observes a much ‘shallower’ quasi-potential in the PMDP model, especially at larger protein numbers (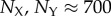). This is due to the fat tails in the exponential bursting kernel of the PDMP model, which are not present in the diffusion approximation of the GB model. Such a fat-tail bursting kernel enhances the probability for the system to evolve to high protein concentrations. We identify this as the origin of the qualitatively distinct rare-event statistics in the two models.

## 5. Bursting noise in a multi-switch network

**Figure 9.**
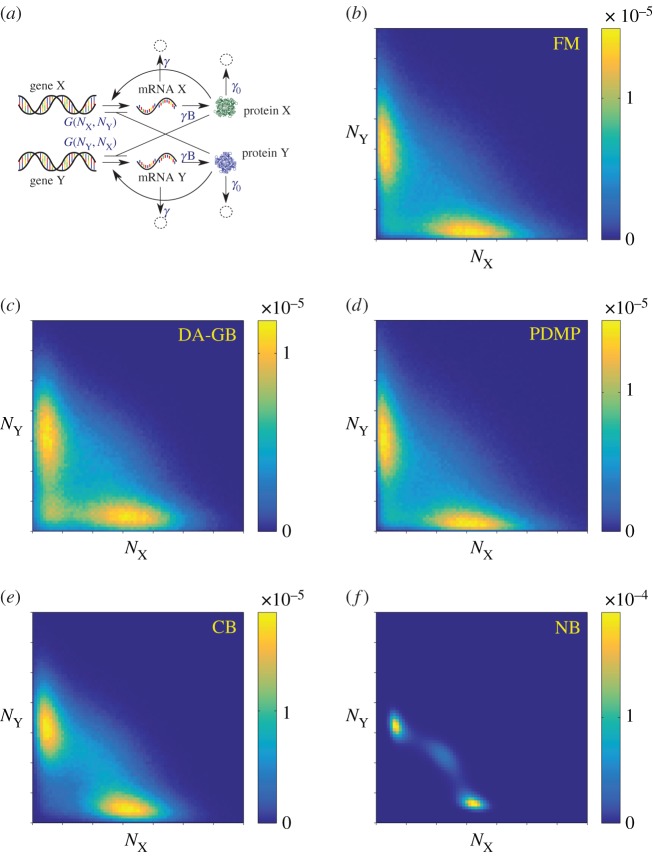
(*a*) Schematic diagram illustrating the network of the three-way switch, remaining panels show stationary distribution of protein numbers in the range 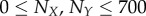 on linear scale. (*b*) Full model; (*c*) diffusion approximation of the GB model; (*d*) PDMP approximation; (*e*) CB model; and (*f*) NB model. (Online version in colour.)

Recently, multi-switch systems have gained interest [13,21,39]. A schematic diagram of the three-way switch network proposed by Lu *et al*. [13] is shown in figure 9*a*. It is obtained from the classical toggle switch network by including a self-enhancing autoregulation. Our computational and mathematical set-up requires only minor modifications to include generalization to this case. Specifically, we replace the earlier Hill functions by
5.1
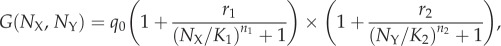
with parameters [13] *q*_0_ = 4, *r*_1_ = −4/5, *r*_2_ = 7/3, *n*_1_ = 3, *n*_2_ = 1, *K*_1_ = 160 and *K*_2_ = 320. The rest of the parameters follow table 1. The negative value of *r*_1_ reflects the positive autoregulation. To evaluate the effects of bursting noise on this multi-switch model, we consider again the FM, the diffusion approximation of the GB model, as well as the CB and NB models of the extended network.

Figure 9 displays the stationary distribution to illustrate the effects of the bursting noise in the multi-switch network. The model without bursts (NB, panel *f*) has a stationary distribution consisting of three modes, as reported earlier [13]. Inclusion of CB (panel *e*) diversifies the protein expression and reduces the stability of the mode located at 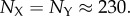 In the FM (panel *b*), there is no discernible concentration of probability in the symmetric mode, hence the three-way switching capability appears to be absent. We also note that the saddle of the distribution in the FM is located at a state with a much lower number of proteins compared with the NB and CB models. The most likely switching path [9] from one of the asymmetric modes to the other will differ significantly between the different variants of the model. The diffusion approximation of the GB model (panel (*c*)) does not capture the outcome of the FM either. Overall, these findings confirm again that the inclusion of bursting noise statistics has significant effects on the model outcome. Finally, we observe in figure 9*d* that the PDMP model approximates the FM of the three-way switch well. We conclude that randomly distributed burst sizes are again the predominant form of intrinsic noise in the multi-switch network.

## 6. Discussion and conclusion

Explicitly including mRNA dynamics in gene regulatory models inevitably introduces more complexity. We have quantitatively studied the effects of bursting noise [1] in a biologically relevant regime of the model organism *E. coli*. To our knowledge, this is one of the first attempts to build a more rigorous connection between existing individual-based models [3,8–10] and coarse-grained models [11,12,14,15].

We have investigated two biologically motivated observables: the stationary distribution of the protein expression and the MFST of the biological switch. The stationary distribution serves as an approximation of the epigenetic distribution of a collection of a large number of similar cells. While the stationary distribution quantifies time-independent properties of the system at long times, the MFST is a measure of the timescale of spontaneous switching. It provides a quantitative characterization of the stability of the switch against intrinsic noise. We note that other quantities, not studied in this work, may be of biological interest as well, for example temporal correlations of fluctuations or indeed the nature of the path taken during switches between states. Our results indicate that the bursting statistics of transcription and translation are essential ingredients of models of gene regulation. Coarse-grained models need to account for bursting to retain correct statistics of noise-driven phenomena such as the switching between different dynamic attractors.

The implications of our observations are relevant to the abstract modelling of regulatory networks in different ways. We are now in a better position to address our opening question and to say how noise propagates between different levels of modelling. Perhaps more importantly, our study may ultimately help to decide what level of modelling is most appropriate to study gene regulatory circuits computationally. The answer will of course depend on the precise nature of the question that is asked. We have examined different levels of coarse graining, and we have identified the steps in these reduction procedures at which significant alternations to different model outcomes are introduced.

Systematically choosing a suitable level of coarse graining also facilitates the mathematical analysis of regulatory networks. The high dimensionality of full regulatory networks effectively makes them intractable. Model reduction is needed to make progress, and our analysis demonstrates that the PDMP formulation is a powerful way forward, and that it can be more suitable than the conventional diffusion approximation. The PDMP model explicitly retains the bursting noise originating from the mRNA dynamics. Even though it effectively disregards the demographic noise from random degradation of the proteins, it delivers accurate predictions for stationary distributions and switching times. We remark that for a lower dimensional system—an autoregulated network—the PDMP approximates the individual-based model as well to a good accuracy and we provide analysis separately [40].

As another strength, the PDMP formulation can be generalized relatively easily to accommodate more complex reactions. For example, in the *Enterobacteria phage λ* switch, it is not the monomer of the synthesized proteins which acts as the repressor to regulate transcription, but instead their dimer. Modelling these processes requires the inclusion of dimerization further downstream after transcription and translation [7,10,26]. Preliminary results not shown here reveal that the PDMP approximates such dynamics well. In addition, the formulation can be generalized to model the switching between genetic states [26,41].

The fact that the PDMP is successful in approximating the FM opens a relatively new type of modelling paradigm. We acknowledge that we are not the first to propose this type of model [41–46]. Our contribution consists of a first analytical treatment of PDMP models in the shot-noise limit 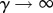 to investigate the bursting nature due to the presence of short-lived mRNA molecules in prokaryotic cells. This is in contrast to the previously cited references which focus on eukaryotic cells. We have also provided a more systematic embedding of PDMP into a wider landscape of modelling approaches.

The bursting phenomenon is ubiquitous whenever there is a separation of timescales between the source and the product of a biological process. These are mRNA and protein in models of gene regulation, but we expect that these ideas can be applied to other biological problems with similar timescale separation.

## Supplementary Material

Supplementary Materials
