# Supplementary material for: Bursting noise in gene expression dynamics: linking microscopic and mesoscopic models
Source: J R Soc Interface. 2016 Jan;13(114):20150772. doi: 10.1098/rsif.2015.0772 (PMC4759790; doi:10.1098/rsif.2015.0772)
Supplement: Supplementary Materials [file rsif20150772supp1.pdf]

Supplementary Information
Bursting noise in gene expression dynamics: Linking microscopic and mesoscopic models

Yen Ting Lin* and Tobias Galla†

Theoretical Physics, School of Physics and Astronomy,

The University of Manchester, Manchester M13 9PL, UK

(Dated: November 30, 2015)

* yenting.lin@manchester.ac.uk

† tobias.galla@manchester.ac.uk

CONTENTS

I. Notation 4

II. Master equations of the individual-based models 4

A. Master equation of full model (FM) 4

B. Infinitely fast-degrading mRNA limit: the GB model 4

C. Master equation of the GB model 5

D. Master equation of CB model 6

E. Master equation of the model without bursts (NB) 6

III. Quantitative comparison of the individual-based models 6

IV. Deriving the diffusion approximations 7

A. Diffusion approximation of the GB model 7

B. Diffusion approximation of the CB and NB models 8

V. The piecewise deterministic Markov process (PDMP) 9

A. Construction of the PDMP model 9

B. Forward equation in the limit $\gamma \to \infty$ 12

C. Equations for the mean first switching time 13

VI. WKB analysis 14

A. WKB ansatz 14

B. DA of the GB model 14

C. DA of the CB model 14

D. DA of the NB model 15

E. PDMP model 15

VII. Numerical methods 15

VIII. Sample paths of the different models 17

A. Full model 17

B. Geometrically distributed burst model (GB) 18

C. Constant burst model (CB) 18

D. No-burst model (NB) 19

E. PDMP model 19

F. Diffusion approximation of the GB model 20
G. Diffusion approximation of the CB model 20
H. Diffusion approximation of the NB model 21

IX. Comparison of stationary distributions 22

X. Comparison of the mean first switching times 23

XI. Comparing WKB results 24

References 25

I. NOTATION

We briefly summarize the notation used in the main manuscript and in this supplement:

- Discrete numbers of the two types of protein are denoted by $N_{\rm X}$ and $N_{\rm Y}$. We write $M_{\rm X}$ and $M_{\rm Y}$ for the number of mRNA molecules of the two types.
- Variables such as $x(t)$ denote continuous particle densities (or concentrations). Specifically $x(t)$ and $y(t)$ are protein densities, i.e., $x = N_{\rm X}/K$ and $y = N_{\rm Y}/K$ in the limit $K \gg 1$.
- We denote the probabilities in the master equations by capital P (discrete particle numbers).
- The lower-case notation p is used for probability density functions in the diffusion approximation (continuous particle densities/concentrations).

II. MASTER EQUATIONS OF THE INDIVIDUAL-BASED MODELS

The different individual-based model in the main manuscript are uniquely defined by their master equations. Here we briefly summarize the master equations of the FM and of the GB, CB and NB models.

A. Master equation of full model (FM)

We write $P_{a,b,c,d}$ for the probability that the system is in state $M_{\rm X} = a, M_{\rm Y} = b, N_{\rm X} = c, N_{\rm Y} = d$ at time t. The master equation of the FM is then

$$\begin{aligned}\frac{d}{dt}P_{a,b,c,d} = & -\left\{H\left(c\right)+H\left(d\right)+a\gamma\left[1+B\right]+b\gamma\left[1+B\right]-\gamma_0 c-\gamma_0 d\right\}P_{a,b,c,d} \\ & +H\left(c\right)P_{a,b-1,c,d}+H\left(d\right)P_{a-1,b,c,d}+\gamma\left(a+1\right)P_{a+1,b,c,d}+\gamma\left(b+1\right)P_{a,b+1,c,d} \\ & +B\gamma a P_{a,b,c-1,d}+B\gamma b P_{a,b,c,d-1}+\gamma_0\left(c+1\right)P_{a,b,c+1,d}+\gamma_0\left(d+1\right)P_{a,b,c,d+1}. \end{aligned} \quad (1)$$

The probability of a state is zero if any of the variables a, b, c or d are negative.

B. Infinitely fast-degrading mRNA limit: the GB model

In the kinetic scheme of the full model the mRNA decays with a rate γ, but synthesizes a protein with a rate γB. Both of the rates are constants. Once an mRNA is created the next event involving this mRNA particle is either the production of protein or the decay of the mRNA molecule. The

probability that the next event is the synthesis of a protein is $B/(B+1)$, and the probability that a decay occurs next (before production of a protein) is $1/(B+1)$. The random number, ℓ, of protein molecules generated by one particular mRNA molecule during its lifetime then follows a geometric distribution

$$g(\ell) = \left(\frac{B}{1+B}\right)^{\ell} \left(\frac{1}{1+B}\right). \tag{2}$$

The lifetime of an mRNA molecule is of order $\mathcal{O}(1/\gamma)$. As a consequence, we can think of the protein-generating process as follows in the infinitely-fast decaying mRNA limit ($\gamma \to \infty$): As soon as an mRNA is transcribed, it immediately releases a random number of proteins ℓ drawn from the distribution (2) and then decays.

C. Master equation of the GB model

In the limit $\gamma \to \infty$, the dynamics of the full model can effectively be coarse-grained into a single-species model

$$\text{Gene X} \xrightarrow{H(N_{\text{Y}})} \ell \times \text{Protein X} \quad \text{(transcription and translation of protein X)}, \tag{3a}$$

$$\text{Gene Y} \xrightarrow{H(N_{\text{X}})} \ell \times \text{Protein Y} \quad \text{(transcription and translation of protein Y)}, \tag{3b}$$

$$\text{Protein X} \xrightarrow{\gamma_0} \emptyset \qquad \text{(degradation of protein X)}, \tag{3c}$$

$$\text{Protein Y} \xrightarrow{\gamma_0} \emptyset \qquad \text{(degradation of protein Y)}, \tag{3d}$$

where ℓ is drawn from the above geometric distribution, every time one of the first two reactions fires. The master equation of this process is

$$\begin{aligned} \frac{d}{dt} P_{c,d} = & - \left[H(c) + H(d) + \gamma_0 c + \gamma_0 d\right] P_{c,d} + \gamma_0 (c+1) P_{c+1,d} + \gamma_0 (d+1) P_{c,d+1} \\ & + \sum_{\ell=0}^{c} H(d) \left(\frac{B}{1+B}\right)^{\ell} \left(\frac{1}{1+B}\right) P_{c-\ell,d} + \sum_{\ell=0}^{d} H(c) \left(\frac{B}{1+B}\right)^{\ell} \left(\frac{1}{1+B}\right) P_{c,d-\ell}. \end{aligned} \tag{4}$$

We have written $P_{c,d}(t)$ for the probability that the system is in state $N_{\text{X}} = c, N_{\text{Y}} = d$ at time t. Again, the probability of a state is zero if c or d are negative.

Model	Jansen–Shannon distance (to FM)	MFST (unit: cell cycle)
Full model (FM)	0	9.9214
Geometric bursting (GB)	1.185×10^{-3}	9.0993
Constant bursting (CB)	3.380×10^{-2}	17.0769
No bursting (NB)	0.360	> 1000

TABLE I. Jensen–Shannon distance between stationary distributions the coarse-grained models (GB, CB, and NB) and the full model (FM), and the mean first switching times of the models when the initial condition is $N_{\mathrm{X}} = 379$ and $N_{\mathrm{Y}} = 89$.

D. Master equation of CB model

The master equation for the CB model is obtained by replacing $g(\ell) \to \delta_{\ell,B}$, i.e., ℓ takes value $\ell = B$ with probability one. We find

$$\begin{aligned}\frac{d}{dt}P_{c,d} = & -\left[H(c) + H(d) + \gamma_0 c + \gamma_0 d\right] P_{c,d} \\ & + H(d)\, P_{c-B,d} + H(c)\, P_{c,d-B} + \gamma_0 (c+1)\, P_{c+1,d} + \gamma_0 (d+1)\, P_{c,d+1}. \end{aligned} \tag{5}$$

E. Master equation of the model without bursts (NB)

In this case we have

$$\begin{aligned}\frac{d}{dt}P_{c,d} = & -\left[BH(c) + BH(d) + \gamma_0 c + \gamma_0 d\right] P_{c,d} \\ & + BH(d)\, P_{c-1,d} + BH(c)\, P_{c,d-1} + \gamma_0 (c+1)\, P_{c+1,d} + \gamma_0 (d+1)\, P_{c,d+1}. \end{aligned} \tag{6}$$

III. QUANTITATIVE COMPARISON OF THE INDIVIDUAL-BASED MODELS

The individual-based models are simulated using standard methods [1, 2]. 10^6 sample paths were simulated for a sufficient long time, and we numerically compute the Jensen–Shannon distance [3, 4] between the resulting stationary distributions of full model (FM) and the coarse-grained protein only models (i.e., GB, CB, and NB). Results are summarized in Table I. In interpreting this data, it is useful to keep in mind that the Jensen–Shannon distance between two distributions P and Q is bounded, $0 \leq \mathrm{JSD}\,(P||Q) \leq \log(2) = 0.6931$.

The MFST of the systems are also measured. We prepare the system with an initial condition $N_{\mathrm{X}} = 371$ and $N_{\mathrm{Y}} = 89$, which is the configuration with integer protein numbers nearest to the fixed point of the mean-field dynamics with the parameter set listed in Table I in the main text. To

measure the mean first switching time 10^5 sample paths were simulated, results are summarised in Table I of this Supplement.

IV. DERIVING THE DIFFUSION APPROXIMATIONS

A. Diffusion approximation of the GB model

Simulations of the full model (FM) show that the number of mRNA molecules present at any one time is typically very small ($M_{\mathrm{X}}, M_{\mathrm{Y}} < 10$) when biologically relevant parameters are used. The conventional diffusion approximation relies on large particle numbers, and so it is not adequate for the full model.

Instead, we perform the diffusion approximation to the master equation of the GB model, equation (4). This is a standard method, and we proceed along the lines of [5]. The only complication is the presence of the geometrically distributed random numbers (denoted by ℓ) in the protein-generation reactions. As we will discuss below this requires only modest modifications to the standard Kramers-Moyal expansion.

We assume that the scale of the population size, K, is large but finite, i.e., $K \gg 1$, and we write $x = N_{\mathrm{X}}/K$ and $y = N_{\mathrm{Y}}/K$, and replace $P_{c,d}(t)$ in favour of $p(x,y,t)$. The master equation (4) then becomes

$$\begin{aligned}\partial_t p(x,y,t) = & -\left[H(Kx) + H(Ky) + \gamma_0 Kx + \gamma_0 Ky\right] p(x,y,t) \\ & + \gamma_0 K\left(x + \frac{1}{K}\right) p\left(x + \frac{1}{K}, y, t\right) + \gamma_0 K\left(y + \frac{1}{K}\right) p\left(x, y + \frac{1}{K}, t\right) \\ & + \sum_{\ell=0}^{\infty} H(Ky)\left(\frac{B}{1+B}\right)^{\ell}\left(\frac{1}{1+B}\right) p\left(x - \frac{\ell}{K}, y, t\right) \\ & + \sum_{\ell=0}^{\infty} H(Kx)\left(\frac{B}{1+B}\right)^{\ell}\left(\frac{1}{1+B}\right) p\left(x, y - \frac{\ell}{K}, t\right). \end{aligned} \tag{7}$$

In the last two terms we have extended the summation over ℓ to infinity, terms in which $x - \ell/K$ or $y - \ell/K$ become negative are automatically suppressed as the corresponding probabilities $p(x - \ell/K, y, t)$ and $p(x, y - \ell/K, t)$ vanish.

The above expression can then be written as

$$\begin{aligned}\partial_t p(x,y,t) = & -\left[H(Kx) + H(Ky) + \gamma_0 Kx + \gamma_0 Ky\right] p(x,y,t) \\ & + \gamma_0 K\left(x + \frac{1}{K}\right) p\left(x + \frac{1}{K}, y, t\right) + \gamma_0 K\left(y + \frac{1}{K}\right) p\left(x, y + \frac{1}{K}, t\right) \\ & + H(Ky)\left\langle p\left(x - \frac{\ell}{K}, y, t\right)\right\rangle_{\ell} + H(Kx)\left\langle p\left(x, y - \frac{\ell}{K}, t\right)\right\rangle_{\ell}, \end{aligned} \tag{8}$$

where $\langle\cdots\rangle_l$ denotes an average with respect to a geometrically distributed random number ℓ, i.e., $\langle f_\ell\rangle_\ell = (1+B)^{-1}\sum_\ell \left(\frac{B}{1+B}\right)^\ell f_\ell$.

We next expand the above equation in powers of $1/K$, keeping only the leading and sub-leading order terms [5]. We also use the explicit expressions $\langle\ell\rangle_\ell = B$ and $\langle\ell^2\rangle_\ell = B(2B+1)$ for the first two moments of the geometric distribution.

We arrive at the Fokker–Planck equation

$$\begin{aligned}\partial_t p(x,y,t) =& -\partial_x\left\{\left[\frac{B}{K}H\left(Ky\right)-\gamma_0 x\right]p\left(x,y,t\right)\right\} - \partial_x\left\{\left[\frac{B}{K}H\left(Kx\right)-\gamma_0 y\right]p\left(x,y,t\right)\right\}\\ &+\frac{1}{2K}\partial_x^2\left\{\left[\frac{B\left(2B+1\right)}{K}H\left(Ky\right)+\gamma_0 x\right]p\left(x,y,t\right)\right\}\\ &+\frac{1}{2K}\partial_y^2\left\{\left[\frac{B\left(2B+1\right)}{K}H\left(Kx\right)+\gamma_0 y\right]p\left(x,y,t\right)\right\},\end{aligned} \tag{9}$$

where we have written $\partial_x = \frac{\partial}{\partial x}$ and similarly for ∂_y. Realizations of the random process described by the Fokker-Planck equation (9) can be obtained as the solutions of the coupled Itō stochastic differential equations

$$dx_t = v(x_t, y_t)dt + \sqrt{D(x_t, y_t)}dW_t^{(x)}, \tag{10a}$$

$$dy_t = v(y_t, x_t)dt + \sqrt{D(y_t, x_t)}dW_t^{(y)}, \tag{10b}$$

with the defined drift v and diffusion D given by

$$v(w,z) := B\left(r_0 + \frac{r}{1+z^n}\right) - \gamma_0 w, \tag{11a}$$

$$D(w,z) := \frac{B}{K}\left[(2B+1)\left(r_0+\frac{r}{1+z^n}\right)+\frac{1}{B}\gamma_0 w\right]. \tag{11b}$$

The quantities $W_t^{(x)}$ and $W_t^{(y)}$ are independent Wiener processes.

B. Diffusion approximation of the CB and NB models

The same procedure can be applied to the master equation of the CB and NB models, and for completeness we report the resulting Fokker-Planck equations.

For the CB model one finds

$$\begin{aligned}\partial_t p(x,y,t) =& -\partial_x\left\{\left[\frac{B}{K}H\left(Ky\right)-\gamma_0 x\right]p\left(x,y,t\right)\right\} - \partial_x\left\{\left[\frac{B}{K}H\left(Kx\right)-\gamma_0 y\right]p\left(x,y,t\right)\right\}\\ &+\frac{B}{2K}\partial_x^2\left\{\left[\frac{B}{K}H\left(Ky\right)+\frac{1}{B}\gamma_0 x\right]p\left(x,y,t\right)\right\}\\ &+\frac{B}{2K}\partial_y^2\left\{\left[\frac{B}{K}H\left(Kx\right)+\frac{1}{B}\gamma_0 y\right]p\left(x,y,t\right)\right\},\end{aligned} \tag{12}$$

and for the model without bursts (NB) one has

$$\partial_t p(x,y,t) = -\partial_x \left\{ \left[\frac{1}{K} BH(Ky) - \gamma_0 x \right] p(x,y,t) \right\} - \partial_x \left\{ \left[\frac{1}{K} BH(Kx) - \gamma_0 y \right] p(x,y,t) \right\} + \frac{1}{2K} \partial_x^2 \left\{ \left[\frac{1}{K} BH(Ky) + \gamma_0 x \right] p(x,y,t) \right\} + \frac{1}{2K} \partial_y^2 \left\{ \left[\frac{1}{K} BH(Kx) + \gamma_0 y \right] p(x,y,t) \right\}. \tag{13}$$

V. THE PIECEWISE DETERMINISTIC MARKOV PROCESS (PDMP)

A. Construction of the PDMP model

In this section we outline the construction of the PDMP approximation, starting from the full model. For this purpose it is useful to introduce the notation

$$P^{(a,b)}(c,d,t) = P(M_{\rm X} = a, M_{\rm Y} = b, N_{\rm X} = c, N_{\rm Y} = d, t). \tag{14}$$

Thus the upper indices (a,b) denote the number of mRNA molecules of either type in the system, and the arguments a, b stand for protein numbers.

The master equation (1) can then be written in matrix form

$$\frac{d}{dt} \begin{bmatrix} P^{(0,0)}(c,d,t) \\ P^{(1,0)}(c,d,t) \\ P^{(0,1)}(c,d,t) \\ \cdots \end{bmatrix} = \mathcal{L}^\dagger \begin{bmatrix} P^{(0,0)}(c,d,t) \\ P^{(1,0)}(c,d,t) \\ P^{(0,1)}(c,d,t) \\ \cdots \end{bmatrix}. \tag{15}$$

We have introduced

$$\mathcal{L}^\dagger := \begin{bmatrix} \mathcal{L}^{\dagger(0,0)} - H(c) - H(d) & \gamma & \gamma & \cdots \\ H(d) & \mathcal{L}^{\dagger(1,0)} - H(c) - H(d) - \gamma & 0 & \cdots \\ H(c) & 0 & \mathcal{L}^{\dagger(0,1)} - H(c) - H(d) - \gamma & \cdots \\ \cdots & & & \end{bmatrix}, \tag{16}$$

where the operator $L^{\dagger(m,n)}$ describes the forward evolution of protein numbers when there are $M_X = m$ and $M_Y = n$ mRNA molecules in the system. Specifically,

$$\mathcal{L}^{\dagger(m,n)} = m\gamma B\left(\mathcal{E}^{-1,0} - 1\right) + n\gamma B\left(\mathcal{E}^{0,-1} - 1\right) + \gamma_0\left[\left(\mathcal{E}^{0,1} - 1\right) + \left(\mathcal{E}^{0,1} - 1\right)\right], \tag{17}$$

where $\mathcal{E}^{i,j}$ are the shift operators [5] acting on functions of protein numbers. They are defined through

$$\mathcal{E}^{i,j} f(m,n) \equiv f(m+i, n+j). \tag{18}$$

Next we consider the limit of fast mRNA decay, that is, large values of γ. More specifically mRNA molecules of either type are generated with rates $H(N_Y)$ and $H(N_X)$ respectively, and we assume that γ is much larger than either of these two rates ($\gamma \gg H(N_Y), \gamma \gg H(N_X)$, for any values of N_X and N_Y). In this limit, the system is almost always in the state without mRNA molecules (*i.e.*, $M_\mathrm{X} = 0, M_\mathrm{Y} = 0$), except for short spells during which there is either one molecule of mRNA of type X, or one of type Y. The duration of the episodes spent in these $(1,0)$ and $(0,1)$ states is of order γ^{-1}, then a switch back to the $(0,0)$ state occurs. The probability to find the system in states with $M_X > 1$ or $M_Y > 1$ is even smaller, specifically of order $(H/\gamma)^2$, and we neglect contributions from these states. Equation (15) can then be simplified into a forward equation of a three-state model:

$$\frac{d}{dt}\begin{bmatrix} P^{(0,0)}(c,d,t) \\ P^{(1,0)}(c,d,t) \\ P^{(0,1)}(c,d,t) \end{bmatrix} = \mathcal{L}^\dagger_\mathrm{approx}\begin{bmatrix} P^{(0,0)}(c,d,t) \\ P^{(1,0)}(c,d,t) \\ P^{(0,1)}(c,d,t) \end{bmatrix} \tag{19}$$

with

$$\mathcal{L}^\dagger_\mathrm{approx} := \begin{bmatrix} \mathcal{L}^{\dagger(0,0)} - H(c) - H(d) & \gamma & \gamma \\ H(d) & \mathcal{L}^{\dagger(1,0)} - \gamma & 0 \\ H(c) & 0 & \mathcal{L}^{\dagger(0,1)} - \gamma \end{bmatrix}. \tag{20}$$

In the next step we consider the limit of large values of K. Formally we take the limit $K \to \infty$. The system can then be described by the protein concentration $x = N_\mathrm{X}/K$ and $y = N_\mathrm{Y}/K$. The corresponding probability distributions in the continuum limit are

$$p_0(x,y) := P^{(0,0)}(Kx, Ky)\,K^2, \tag{21a}$$

$$p_\mathrm{X}(x,y) := P^{(1,0)}(Kx, Ky)\,K^2, \tag{21b}$$

$$p_\mathrm{Y}(x,y) := P^{(0,1)}(Kx, Ky)\,K^2. \tag{21c}$$

On the left-hand side we have introduced the notation $0, X$ and Y to describe the states in which there are no mRNA molecules ($M_X = M_Y = 0$), one mRNA molecule of type X ($M_X = 1, M_Y = 0$) and one mRNA molecule of type Y respectively ($M_X = 0, M_Y = 1$). This is in-line with the notation in the main manuscript.

The time evolution of the protein concentrations between the random switching events is taken to be deterministic. Mathematically this corresponds to expanding the discrete operators $\mathcal{L}^{\dagger(a,b)}$ in powers of K^{-1}, and keeping only the lowest-order advection terms. This generates so-called Liouville operators, and leads to

$$\frac{\partial}{\partial t}\begin{bmatrix} p_0 \\ p_\mathrm{X} \\ p_\mathrm{Y} \end{bmatrix} = \left(L_d^\dagger + L_\mathrm{s}^\dagger\right)\begin{bmatrix} p_0 \\ p_\mathrm{X} \\ p_\mathrm{Y} \end{bmatrix}, \tag{22}$$

where $L_d^\dagger$ and $L_s^\dagger$ are the forward operators driving the deterministic flow and the random switching between states, respectively. They are given by

$$L_d^\dagger := \begin{bmatrix} \left(L_d^\dagger\right)_{11} & 0 & 0 \\ 0 & \left(L_d^\dagger\right)_{22} & 0 \\ 0 & 0 & \left(L_d^\dagger\right)_{33} \end{bmatrix}, \tag{23a}$$

$$L_s^\dagger := \begin{bmatrix} -H(Kx) - H(Ky) & \gamma & \gamma \\ H(Ky) & -\gamma & 0 \\ H(Kx) & 0 & -\gamma \end{bmatrix}, \tag{23b}$$

and

$$\left(L_d^\dagger\right)_{11} := \gamma_0 \partial_x\left(x\right) + \gamma_0 \partial_y\left(y\right), \tag{24a}$$

$$\left(L_d^\dagger\right)_{22} := \partial_x\left(-\gamma b + \gamma_0 x\right) + \gamma_0 \partial_y\left(y\right), \tag{24b}$$

$$\left(L_d^\dagger\right)_{33} := \gamma_0 \partial_x\left(x\right) + \partial_y\left(-\gamma b + \gamma_0 y\right). \tag{24c}$$

Nature of the approximation

In deriving Eq. (22) we have made several assumptions and approximations:

(i) First, we have assumed that $\gamma/H \gg 1$, where H stands for the maximum value $H(N_\mathrm{X})$ and $H(N_\mathrm{Y})$ can attain. We recall that $H(N) = K\left[r_0 + \frac{r}{1+(N/K)^h}\right]$. The function $h(x) = r_0 + r/(1+x^n)$ does not involve K or γ, and its maximum value is $r_0 + r$. In dimensionless units, the assumption $\gamma/H \gg 1$ is thus fulfilled if $\gamma \gg (r_0 + r)K$.

(ii) We have replaced the discrete operators $L^{(a,b)}$ by deterministic Liouville operators, i.e., we neglected *demographic stochasticity* of the protein degradation. The purpose of this is to isolate the contribution of the bursting noise, originating from the random switching of the mRNA state $(0, X$ and $Y)$. Making the deterministic approximation for the protein concentrations is formally valid only in the limit of very large protein populations, $K \gg 1$ (K sets the scale of the numbers of protein molecules).

In summary we assume $\gamma \gg (r_0 + r)K$ and $K \gg 1$. We expect our approximations to be accurate when both of these are fulfilled, in particular the typical value of γ above which our theory can be

expected to be accurate will depend on the choice of K, which in turn must be chosen large enough to justify the deterministic approximation of the protein dynamics.

The data in the main manuscript reveals that the mathematical approximation agrees well with simulations for $\gamma = 30$ and $K = 200$. In our simulations we use $r_0 \approx 0.007$ and $r = 0.06$.

B. Forward equation in the limit $\gamma \to \infty$

We start from

$$\partial_t p_0 = \left[\gamma_0 \partial_x x + \gamma_0 \partial_y y - H(Kx) - H(Ky)\right] p_0 + \gamma p_{\mathrm{X}} + \gamma p_{\mathrm{Y}}, \tag{25a}$$

$$\partial_t p_{\mathrm{X}} = \left[\partial_x \left(\gamma_0 x - \gamma b\right) + \gamma_0 \partial_y - \gamma\right] p_{\mathrm{X}} + H\left(Ky\right) p_0, \tag{25b}$$

$$\partial_t p_{\mathrm{Y}} = \left[\partial_y \left(\gamma_0 y - \gamma b\right) + \gamma_0 \partial_x - \gamma\right] p_{\mathrm{Y}} + H\left(Kx\right) p_0. \tag{25c}$$

Applying the operator $\left[\partial_y \left(\frac{\gamma_0}{\gamma} y - b\right) + \frac{\gamma_0}{\gamma}\partial_x - 1\right] \left[\partial_x \left(\frac{\gamma_0}{\gamma} x - b\right) + \frac{\gamma_0}{\gamma}\partial_y - 1\right]$ to both sides of equation (25a) results in

$$\begin{aligned}
&\partial_t \left[\partial_y \left(\frac{\gamma_0}{\gamma} y - b\right) + \frac{\gamma_0}{\gamma}\partial_x - 1\right] \left[\partial_x \left(\frac{\gamma_0}{\gamma} x - b\right) + \frac{\gamma_0}{\gamma}\partial_y - 1\right] p_0 \\
&= \left[\partial_y \left(\frac{\gamma_0}{\gamma} y - b\right) + \frac{\gamma_0}{\gamma}\partial_x - 1\right] \left[\partial_t p_{\mathrm{X}} - H\left(Ky\right) p_0\right] + \left[\partial_x \left(\frac{\gamma_0}{\gamma} x - b\right) + \frac{\gamma_0}{\gamma}\partial_y - 1\right] \left[\partial_t p_{\mathrm{Y}} - H\left(Kx\right) p_0\right] \\
&\quad + \left[\partial_y \left(\frac{\gamma_0}{\gamma} y - b\right) + \frac{\gamma_0}{\gamma}\partial_x - 1\right] \left[\partial_x \left(\frac{\gamma_0}{\gamma} x - b\right) + \frac{\gamma_0}{\gamma}\partial_y - 1\right] \left[\gamma_0 \partial_x x + \gamma_0 \partial_y y - H(Kx) - H(Ky)\right] p_0.
\end{aligned} \tag{26}$$

We note that this equation is not closed in p_0.

Next, we take the $\gamma \to \infty$ limit, keeping in mind that H and γ_0 are finite. The system then almost-surely stays in the 0-state, and consequently $p_{\mathrm{X}}, p_{\mathrm{Y}} \to 0$. Equation (26) then reduces to

$$\partial_t \left(-b\partial_y - 1\right)\left(-b\partial_x - 1\right) p_0 = \left(b\partial_y + 1\right)\left[H\left(Ky\right) p_0\right] + \left(b\partial_x + 1\right)\left[H\left(Kx\right) p_0\right]. \tag{27}$$

The inverse operator of $1 + b\partial_z$ is

$$(1 + b\partial_z)^{-1} f(z) = \int^z \frac{e^{-\frac{z-z'}{b}}}{b} f(z') dz, \tag{28}$$

and so equation (27) turns into the 'forward equation' presented in the main text:

$$\begin{aligned}
\partial_t p_0 = &\ \partial_x \left(\gamma_0 x p_0\right) + \partial_y \left(\gamma_0 y p_0\right) - \left[H(Kx) + H(Ky)\right] p_0 \\
&+ H(Ky) \int_0^x \frac{1}{b} e^{-\frac{x-x'}{b}} p_0\left(x', y, t\right) dx' + H(Kx) \int_0^y \frac{1}{b} e^{-\frac{y-y'}{b}} p_0\left(x, y', t\right) dy'.
\end{aligned} \tag{29}$$

C. Equations for the mean first switching time

Here we illustrate the detailed derivation to the adjoint equation in the main text. We focus on initial conditions $y > x$ and our goal is to calculate the mean time it takes the dynamics to reach states with $x = y$. We write $T_Z(x, y)$ for the time it takes the dynamics to reach a state in which $x = y$ if started from initial condition x, y, and in mRNA state $\mathrm{Z} \in \{0, \mathrm{X}, \mathrm{Y}\}$. The $T_\mathrm{Z}(x, y)$ then satisfy the following adjoint equation [5]

$$-\begin{bmatrix} 1 \\ 1 \\ 1 \end{bmatrix} = (L_d + L_s) \begin{bmatrix} T_0(x,y) \\ T_\mathrm{X}(x,y) \\ T_\mathrm{Y}(x,y) \end{bmatrix}, \tag{30}$$

where L_d and L_s are the adjoint operators of $L_d^\dagger$ and $L_s^\dagger$. They are given by

$$L_d := \begin{bmatrix} (L_d)_{11} & 0 & 0 \\ 0 & (L_d)_{22} & 0 \\ 0 & 0 & (L_d)_{33} \end{bmatrix} \text{ and } L_s = \begin{bmatrix} -H(Kx) - H(Ky) & H(Ky) & H(Kx) \\ \gamma & -\gamma & 0 \\ \gamma & 0 & -\gamma \end{bmatrix}, \tag{31}$$

with

$$(L_d)_{11} = -\gamma_0 x \partial_x - \gamma_0 y \partial_y, \tag{32a}$$

$$(L_d)_{22} = (\gamma b - \gamma_0 x)\,\partial_x - \gamma_0\,(y)\,\partial_y, \tag{32b}$$

$$(L_d)_{33} = -\gamma_0 x \partial_x + (\gamma b - \gamma_0 y)\,\partial_y. \tag{32c}$$

In the infinitely fast degrading mRNA limit, $\gamma \to \infty$, equations (30) can be seen to converge to

$$-\begin{bmatrix} 1 \\ 0 \\ 0 \end{bmatrix} = \begin{bmatrix} -\gamma_0 x\partial_x - \gamma_0 y \partial_y - H(Kx) - H(Ky) & H(Ky) & H(Kx) \\ 1 & b\partial_x - 1 & 0 \\ 1 & 0 & b\partial_y - 1 \end{bmatrix} \cdot \begin{bmatrix} T_0(x,y) \\ T_\mathrm{X}(x,y) \\ T_\mathrm{Y}(x,y) \end{bmatrix}, \tag{33}$$

The boundary conditions for the mean first exist times are determined by $T_\mathrm{Z}(x_b, y_b) = 0$, for all locations $(x_b, y_b) \in \partial\Omega$ at which the deterministic flow driven by $L_d^\dagger$ flows *out of the domain* Ω in state Z. Next, we specify a bounded domain $\Omega_C := \{(x, y) : 0 < x < y, y < C\}$. The boundary conditions of equations (33) are then $T_\mathrm{X}(z, z) = 0$ and $T_\mathrm{Y}(z, C) = 0\ \forall z < C$. We now use these boundary conditions, and integrate the second and the third components of the expression in equation (33). Subsequently we send $C \to \infty$ and arrive at

$$\begin{aligned} -1 = {} & [-\gamma_0 x\partial_x - \gamma_0 y\partial_y - H(Kx) - H(Ky)]\, T_0(x,y) \\ & + H(Ky) \int_x^y \frac{e^{-\frac{x'-x}{b}}}{b} T_0(x', y)\, dx' + H(Kx) \int_y^\infty \frac{e^{-\frac{y'-y}{b}}}{b} T_0(x, y')\, dx'. \end{aligned} \tag{34}$$

VI. WKB ANALYSIS

A. WKB ansatz

In order to find the quasi-stationary distribution of the PDMP model and of the diffusion approximation of the GB and CB models, one uses the ansatz

$$p_{\text{stat}} = \exp\left\{-\frac{1}{\epsilon}\left[S_0\left(x,y\right)+\mathcal{O}\left(\frac{B}{K}\right)\right]\right\} \tag{35}$$

where $\epsilon \propto K^{-1}$ is the magnitude of the intrinsic noise in the protein dynamics. For the purposes of the WKB analysis the noise is assumed to be weak, i.e., $\epsilon \ll 1$.

B. DA of the GB model

In the context of the diffusion approximation of the GB model we use $\epsilon = B/K$. To leading order ($\mathcal{O}\left(K^0\right)$) one finds a Hamilton–Jacobi equation of the form

$$0 = \frac{1}{2}\left(\nabla S_0\right)^{\mathbf{T}}\mathbf{D}\left(\nabla S_0\right)+\mathbf{v}^{\mathbf{T}}\cdot\nabla S_0. \tag{36}$$

The vector $\mathbf{v}$ denotes the deterministic flow

$$\mathbf{v}\left(x,y\right) := \begin{bmatrix} \frac{B}{K}H\left(y\right)-\gamma_0 x \\ \frac{B}{K}H\left(x\right)-\gamma_0 y \end{bmatrix}, \tag{37}$$

and the (scaled) diffusion matrix $\mathbf{D}$ is given by

$$\mathbf{D}\left(x,y\right) := \begin{bmatrix} D_{11}\left(x,y\right) & 0 \\ 0 & D_{22}\left(x,y\right) \end{bmatrix}, \tag{38}$$

with entries

$$D_{11}\left(x,y\right) = \frac{2B+1}{K}H(Ky)+\frac{1}{B}\gamma_0 x, \tag{39a}$$

$$D_{22}\left(x,y\right) = \frac{2B+1}{K}H(Kx)+\frac{1}{B}\gamma_0 y. \tag{39b}$$

C. DA of the CB model

As before we use $\epsilon = B/K$. A similar leading-order calculation delivers the Hamilton–Jacobi equation, which is again of the form described in equation (36). The only differences are minor modifications in the diffusion matrix, which now has entries

$$D_{11}\left(x,y\right) = \frac{B}{K}H(Ky)+\frac{1}{B}\gamma_0 x, \tag{40a}$$

$$D_{22}\left(x,y\right) = \frac{B}{K}H(Kx)+\frac{1}{B}\gamma_0 y. \tag{40b}$$

D. DA of the NB model

It is now convenient to use $\epsilon = 1/K$. Again one finds a Hamilton–Jacobi equation of the form as above. The diffusion matrix now has entries

$$D_{11}(x,y) = \frac{B}{K} H(Ky) + \gamma_0 x, \tag{41a}$$

$$D_{22}(x,y) = \frac{B}{K} H(Kx) + \gamma_0 y. \tag{41b}$$

E. PDMP model

For the PDMP model, similar calculations deliver the Hamilton–Jacobi equation

$$\begin{aligned} 0 = & \left[\gamma_0 x - \frac{B}{K} H(Ky)\right] \partial_x S_0 + \left[\gamma_0 y - \frac{B}{K} H(Kx)\right] \partial_y S_0 \\ & + \left[\gamma_0 x + \gamma_0 y - \frac{B}{K} H(Kx) - \frac{B}{K} H(Ky)\right] (\partial_x S_0)(\partial_y S_0) \\ & + \gamma_0 x (\partial_x S_0)^2 + \gamma_0 y (\partial_y S_0)^2 + \gamma_0 x (\partial_x S_0)^2 (\partial_y S_0) + \gamma_0 y (\partial_x S_0)(\partial_y S_0)^2 . \end{aligned} \tag{42}$$

VII. NUMERICAL METHODS

Sample paths of the individual-based processes (FM, CB, NB, and GB) are generated by the standard kinetic Monte Carlo algorithm [1, 2] implemented in c++. The PDMP process is simulated using the algorithm proposed by Bokes et al [6]. Simulations of the diffusion approximations are performed using the standard Euler–Maruyama algorithm with a constant time step $\delta t = 10^{-4}/K$. In all cases 10^6 sample paths are simulated for a sufficiently long time to measure stationary distributions. For the mean first switching times, we sample 105 initial states on a lattice on the domain $0 \leq N_{\mathrm{X}}(0) < N_{\mathrm{Y}}(0) \leq 700$. For each initial state, we simulate 10^4 sample paths, each until they cross the boundary $N_{\mathrm{X}} = N_{\mathrm{Y}}$ to measure the mean first switching times.

The geometric minimum action method proposed by Heymann and Vanden–Eijnden [7] is implemented using MATLAB R2010a, and used to find the quasi-potential S_0 of the WKB method. For each model, we sample at least 150 end points and solve for the least-action paths, discretized into 257 equidistant points, connecting one of the fixed points and the end point. The final landscapes are generated by linear interpolation of the quasi-potential so obtained.

The finite-difference scheme to solve the adjoint equation was implemented in MATLAB R2010a, discretizing the domain $0 \leq x, y \leq C = 2000$ into 150×150 grid points. The adjoint equation is then transformed to a set of 22500 linear equations, which is solved using a built-in numerical solver in

MATLAB R2010a.

VIII. SAMPLE PATHS OF THE DIFFERENT MODELS

A. Full model

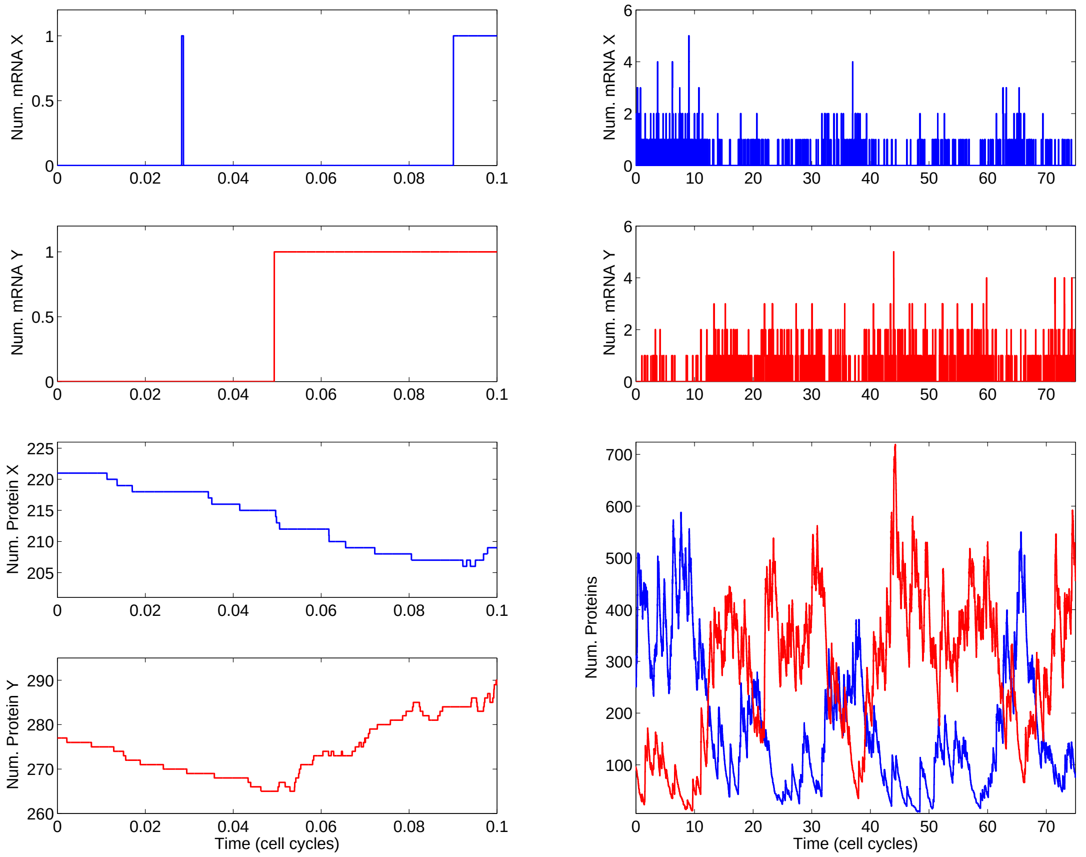

FIG. 1. One sample path of the full model (FM). Left-hand panel: short time scale. Right-hand panel: the protein expressions switches at a longer time scale.

B. Geometrically distributed burst model (GB)

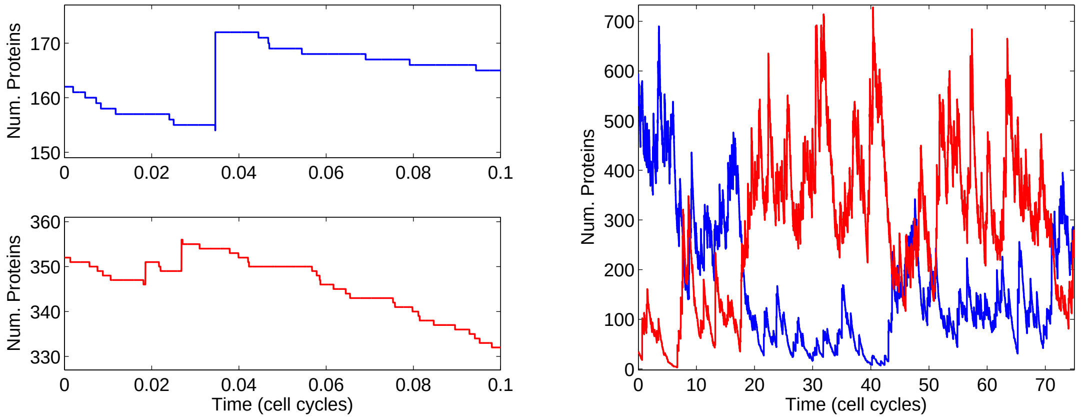

FIG. 2. One sample path of the model with geometrically distributed burst size (GB). Left-hand panel: short time scale. Right-hand panel: long time scale.

C. Constant burst model (CB)

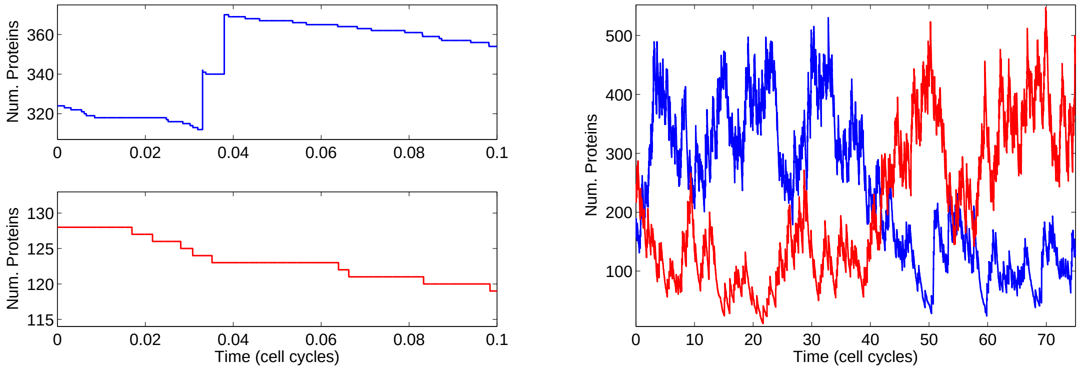

FIG. 3. One sample path of the model with constant bursts (CB). Left-hand panel: short time scale. Right-hand panel: long time scale.

D. No-burst model (NB)

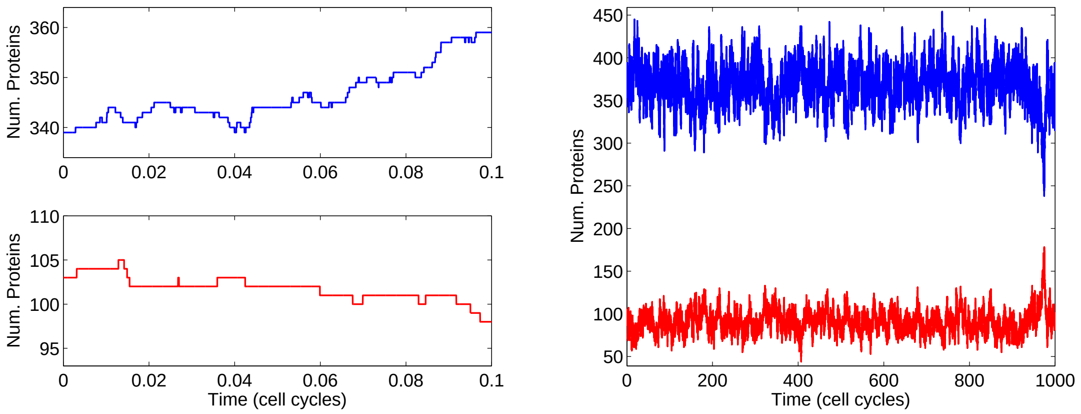

FIG. 4. One sample path of the model without bursts. Left-hand panel: short time scale. Right-hand panel: long time scale. In 1000 cell cycles, we observe no switching event in this sample path.

E. PDMP model

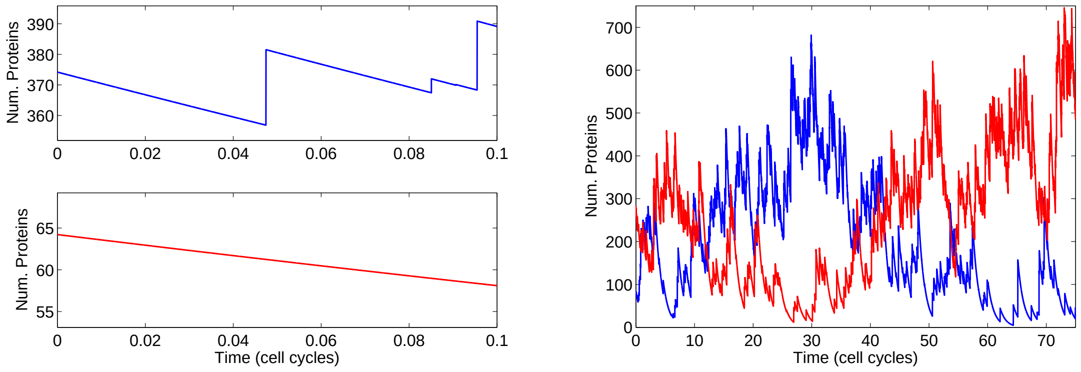

FIG. 5. One sample path of the PDMP. Left-hand panel: short time scale. Right-hand panel: long time scale.

F. Diffusion approximation of the GB model

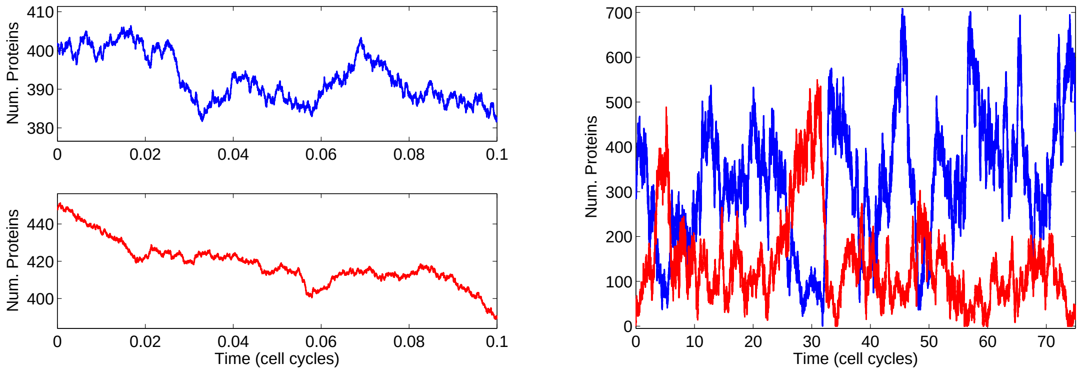

FIG. 6. One sample path of the diffusion approximation of the GB model. Left-hand panel: short time scale. Right-hand panel: long time scale.

G. Diffusion approximation of the CB model

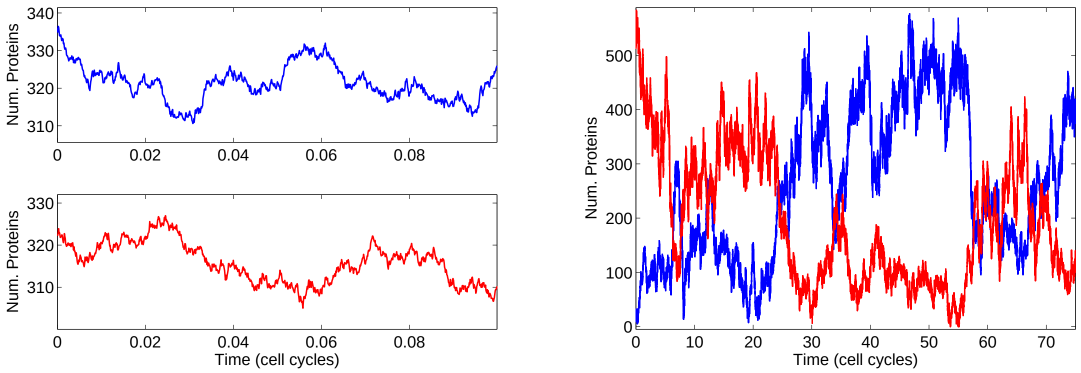

FIG. 7. One sample path of the diffusion approximation of the CB model. Left-hand panel: short time scale. Right-hand panel: long time scale.

H. Diffusion approximation of the NB model

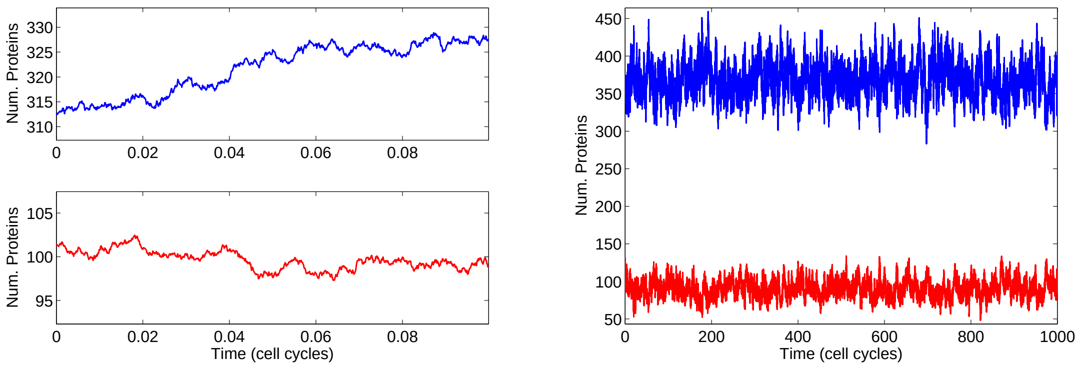

FIG. 8. One sample path of the diffusion approximation of the single-stage model without bursts. Left-hand panel: short time scale. Right-hand panel: long time scale. Similar to the NB model, no switching event occurs in 1000 cell cycles in this sample path.

IX. COMPARISON OF STATIONARY DISTRIBUTIONS

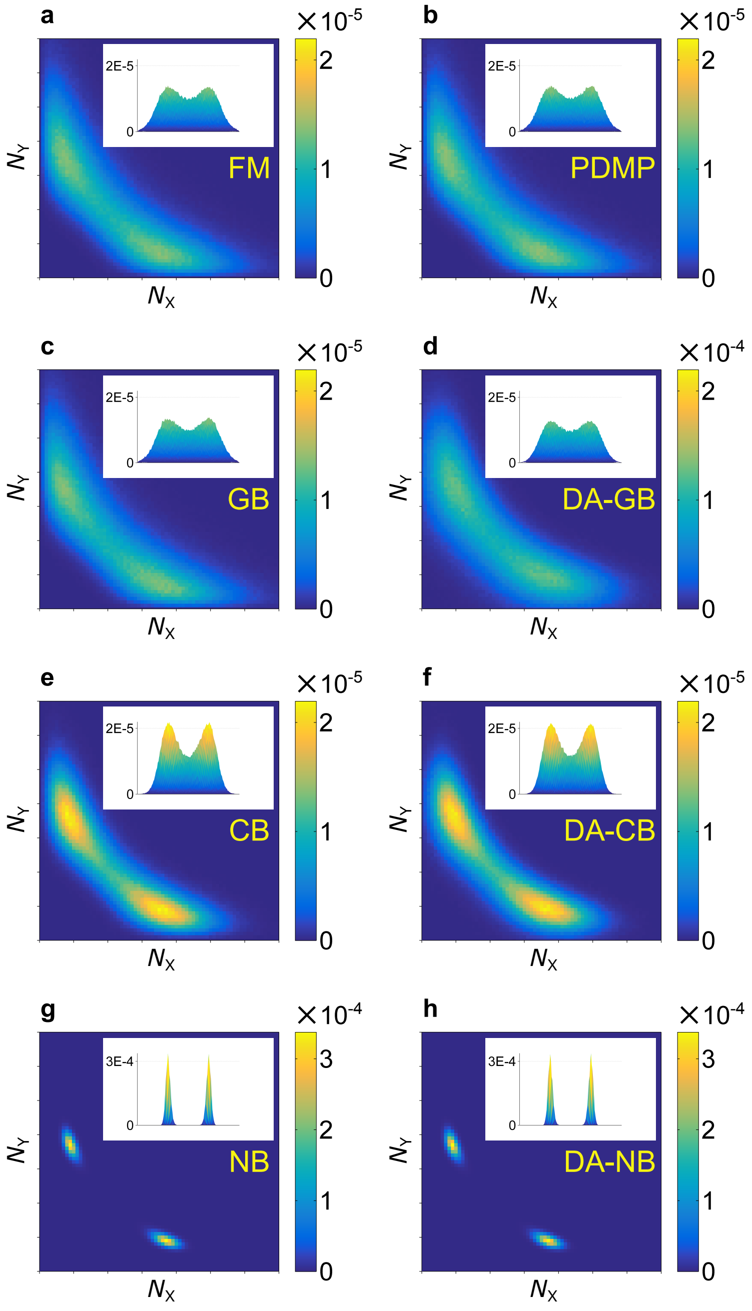

FIG. 9. Stationary distribution measured in simulations. All axes show $0 \leq N_X, N_Y \leq 700$ on a linear scale. Insets show the distribution as viewed from the point $N_\mathrm{X} = N_\mathrm{Y} = 700$ facing towards the origin. (**a**) Full model; (**b**) PDMP; (**c**) GB model; (**d**) Diffusion approximation (DA) of GB; (**e**) CB model; (**f**) DA of CB; (**g**) NB model; (**a**) DA of NB. The same colour scale is used in all panels, except for panels e and f.

X. COMPARISON OF THE MEAN FIRST SWITCHING TIMES

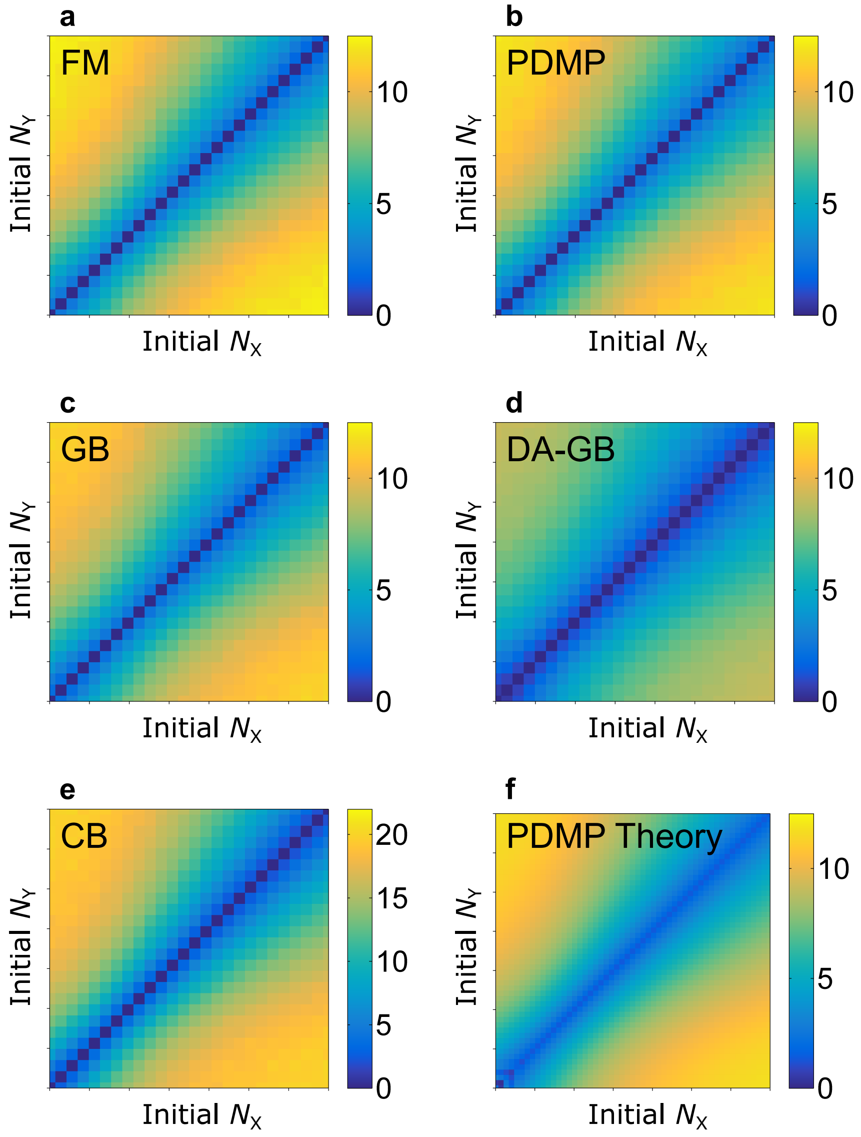

FIG. 10. Mean first switching times. All graphs show $0 \leq N_X, N_Y \leq 700$ on linear scales. (**a**) Full model; (**b**) PDMP; (**c**) GB model; (**d**) Diffusion approximation (DA) of GB; (**e**) CB model; (**f**) Numerical solution of the adjoint equation of the PDMP. Data are plotted on the same colour scale in all panels except for the CB model.

XI. COMPARING WKB RESULTS

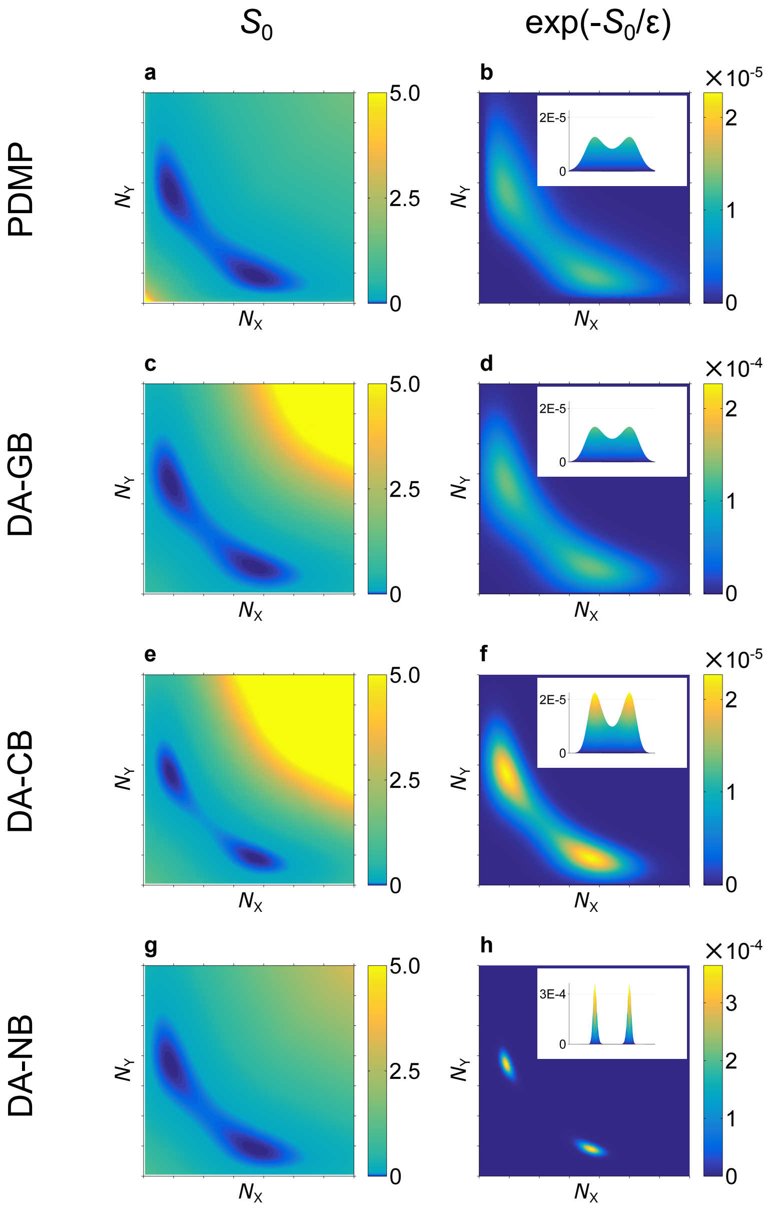

FIG. 11. Results from the WKB analysis. Panels a, c, e, and g show the WKB quasi-potentials $S_0(N_X, N_Y)$, and panels b, d, f, and h the corresponding approximation for the stationary probability distribution $\mathcal{N} \exp\left[-S_0\left(N_\mathrm{X}, N_\mathrm{Y}\right)/\epsilon\right]$ where $\mathcal{N}$ is the normalization factor. All panels show $0 \leq N_X, N_Y \leq 700$ on a linear scale. The insets show the stationary distributions viewed from $(N_\mathrm{X}, N_\mathrm{Y}) = (700, 700)$. (**a, b**) PDMP; (**c, d**) DA of GB ; (**e, f**) DA of CB; (**g, h**) DA of NB.

[1] Schwartz R. 2008 Biological Modeling and Simulation : A Survey of Practical Models, Algorithms, and Numerical Methods. Cambridge: MIT Press.

[2] Gillespie DT. 1977 Exact stochastic simulation of coupled chemical-reactions. *J Phys Chem* **81**, 2340–2361.

[3] Lin JH. 1991 Divergence measures based on the Shannon entropy. *IEEE Trans Inf Theory* **37**, 145–151.

[4] Endres DM, Schindelin JE. 2003 A new metric for probability distributions. *IEEE Trans Inf Theory* **49**, 1858–1860.

[5] van Kampen NG, VanKampen NG. 2007 Stochsatic Processes in Physics and Chemistry. Amsterdam: Elsevier Science B.V.

[6] Bokes P, King JR, Wood ATA, Loose M. 2013 Transcriptional bursting diversifies the behaviour of a toggle switch: Hybrid simulation of stochastic gene expression. *Bull Math Biol* **75**, 351–371.

[7] Heymann M, Vanden-Eijnden E. 2008 The geometric minimum action method: A least action principle on the space of curves. *Comm Pure Appl Math* **61**, 1052–1117.
